# Choice architecture promotes sustainable choices in online food-delivery apps

**DOI:** 10.1093/pnasnexus/pgae422

**Published:** 2024-09-19

**Authors:** Paul M Lohmann, Elisabeth Gsottbauer, James Farrington, Steve Human, Lucia A Reisch

**Affiliations:** El-Erian Institute of Behavioural Economics and Policy, Judge Business School, University of Cambridge, Trumpington Street, Cambridge CB2 1AG, United Kingdom; Free University of Bozen-Bolzano, Competence Center of Economic, Ecological and Social Sustainability, 39100 Bozen-Bolzano, Italy; Institute of Public Finance, Department of Economics and Statistics, University of Innsbruck, A-6020 Innsbruck, Austria; Grantham Research Institute for Climate Change and the Environment, The London School of Economics and Political Science, London WC2A 3PH, United Kingdom; Behavioural Insights Team, London EC4Y 0DS, United Kingdom; Behavioural Insights Team, London EC4Y 0DS, United Kingdom; El-Erian Institute of Behavioural Economics and Policy, Judge Business School, University of Cambridge, Trumpington Street, Cambridge CB2 1AG, United Kingdom

**Keywords:** carbon-footprint labeling, choice architecture, food-delivery apps, low-carbon diets, repositioning

## Abstract

Greenhouse gas emissions from the food system constitute about one-third of the global total, hence mitigation in this sphere of human activity is a vital goal for research and policy. This study empirically tests the effectiveness of different interventions to reduce the carbon footprint of food choices made on food-delivery apps, using an incentive-compatible online randomized controlled trial with 4,008 participants. The experiment utilized an interactive web platform that mimics popular online food-delivery platforms (such as Just Eat) and included three treatment conditions: a sign-posted meat tax, a carbon-footprint label, and a choice-architecture intervention that changed the order of the menu so that the lowest carbon-impact restaurants and dishes were presented first. Results show that only the choice-architecture nudge significantly reduced the average meal carbon footprint—by 0.3 kg/CO_2_e per order (12%), driven by a 5.6 percentage point (13%) reduction in high-carbon meal choices. Moreover, we find evidence of significant health and well-being co-benefits. Menu repositioning resulted in the average meal order having greater nutritional value and fewer calories, whilst significantly increasing self-reported satisfaction with the meal choice. Simple back-of-the-envelope calculations suggest that menu repositioning would be a highly cost-effective policy instrument if implemented at scale, with the return on investment expected to be in the range of £1.28 to £3.85 per metric ton of avoided CO_2_ emissions, depending on implementation costs.

**Significance Statement**

Encouraging healthy and sustainable diets is a priority for climate and health policies. Food-delivery platforms have become more prominent in recent years and offer unique opportunities to design user interfaces that encourage customers to choose meals that are healthy and have a smaller carbon footprint. We conducted an incentivized experiment on a simulated food-delivery app to test three potential food-policy interventions: carbon labels, a meat tax, and placing the most sustainable option at the top of the menu. We find that repositioning menus in order of sustainability is highly effective in encouraging healthier and more sustainable food choices without compromising customers’ choice satisfaction. We estimate that menu repositioning would be a highly cost-effective policy instrument to promote low-carbon food choices.

## Introduction

Addressing greenhouse gas emissions from the global food system, which account for approximately one-third of all emissions, is a primary focus for both research and policy initiatives. This effort is steering the transition from high-carbon, meat-centric diets to more sustainable, plant-based choices (1–4). To effectively guide consumers towards more sustainable food choices, it is necessary to thoroughly explore and experiment with demand-side interventions across different decision contexts. Previous research focused predominantly on interventions in grocery retail and cafeteria settings, including information provision and education (5, 6), price changes (7), carbon-footprint labeling (8–12), and various choice-architecture techniques (13–20). While important, studying interventions in isolation within limited decision settings gives us an incomplete picture. Moreover, the food-choice landscape has seen the emergence and growing popularity of food-delivery apps, which have received limited scrutiny to date (21–25). Food-delivery apps and websites offer unparalleled opportunities for promoting sustainability, as platforms can seamlessly integrate menu design changes and price adaptations, as well as provide (emissions) information at the point of purchase (26–28). Hence, developing and testing interventions uniquely tailored to food-delivery apps offers a compelling strategy to accelerate sustainable dietary change.

This paper contributes to our understanding of how price and nonprice interventions influence consumers’ food choices on app-based food-delivery platforms. Price interventions, such as taxes and subsidies, alter consumer incentives by adjusting the relative prices of sustainable and less sustainable options. In contrast, nonprice interventions, such as menu repositioning and labeling, influence choices through choice architecture and salience, or by providing information, without changing prices. Both types of interventions are considered promising for guiding food choices (29). Our approach thus focused on comparing these interventions to identify the most effective strategy for encouraging sustainable food choices on food-delivery apps. In a preregistered incentive-compatible experiment, we recruited a large sample (*N* = 4,008) representative of the UK internet-using population and asked them to place a meal order on a simulated food-delivery app closely resembling popular food-delivery services such as Uber Eats or Deliveroo. Specifically, these interventions include the implementation of a meat tax, which adjusts meal prices based on the carbon content of their meat ingredients, the use of carbon-footprint labels to provide information on the climate impact, and a choice-architecture intervention that reorders the menu layout to promote low-carbon options. We hypothesized that all three interventions would lead to lower consumption of meat and high-carbon dishes, resulting in an overall reduction in emissions from food orders. In addition, we were able to quantify the potential health and well-being implications of environmentally motivated interventions, including health co-benefits and hedonic well-being effects. Drawing on a range of individual-level socio-demographic and attitudinal data, further exploratory analysis allowed us to identify segments of the population most receptive to the interventions and most inclined to support their implementation on real-world food-delivery platforms, providing valuable insights into sample heterogeneity (30). Assessing intervention effectiveness alongside the impacts on choice satisfaction and public backing provides a comprehensive perspective on the feasibility and potential impact of these measures. Our findings offer insights for policymakers exploring diverse regulatory approaches for food-delivery platforms and present opportunities for service providers to voluntarily contribute to sustainable and healthy diets. Moreover, choice-architecture interventions, such as repositioning environmentally friendly meals first, could serve as a convincing business strategy for online food platforms facing increasing pressure to demonstrate corporate responsibility towards climate and public health, and thus adopting such interventions can enhance their social “licence to operate” (30). At the same time, by prioritizing sustainable food options, these platforms will also align with growing consumer expectations for sustainable food options.

## Experimental design and data

We conducted one of the first incentive-compatible online randomized controlled trials on interventions to promote sustainable food choices on food-delivery apps. The experiment was preregistered via OSF (https://osf.io/h47yj), and all deviations from the preregistration are recorded in Supplementary material, Appendix Section 2.8. We recruited an online representative sample of 4,008 adult consumers in the UK and asked them to complete a food-choice task on a platform resembling popular delivery apps. The platform included nine restaurants that were based on real-world equivalents and offered a variety of popular cuisines. Each restaurant’s menu included a selection of starters, mains, desserts, and drinks (if applicable), to make the choice environment as realistic as possible. In total, participants could select from 164 unique food items for which we calculated the carbon footprint and calorie content. Prices were based on market prices and adjusted to enable any combination of two items (one main and one additional item) to be purchased within a £20 budget in any of the conditions. A detailed description of the menu composition and platform calibration is provided in Supplementary material, Appendix Section 2.1.

Each participant accessing the platform was randomly assigned to one of four conditions: (i) the control condition consisted of a “business as usual” version of the platform (*N* = 990); (ii) the price condition changed food prices proportionally to the carbon content of meat items, resulting in an average increase in the price of meat items by 10%, equivalent to a carbon price of £483 per tonne of CO_2_e (*N* = 1,015). This 10% increase was considered a realistic scenario for introducing a meat tax. Moreover, the tax was clearly sign-posted through a red “T” icon to ensure visibility, and both the selection pop-up and checkout basket included an extra row showing the price increase labeled as meat tax; (iii) the information condition added data on the carbon footprint of food items through standardized carbon-footprint labels with traffic-light color coding (*N* = 994); and (iv) the choice-architecture condition utilized a repositioning technique whereby restaurants and menus were presented in descending order of sustainability, and the lowest-carbon options were shown first (*N* = 1,009). In conditions (i) to (iii), the display order of restaurants and menu items was randomized.

**Fig. 1.**
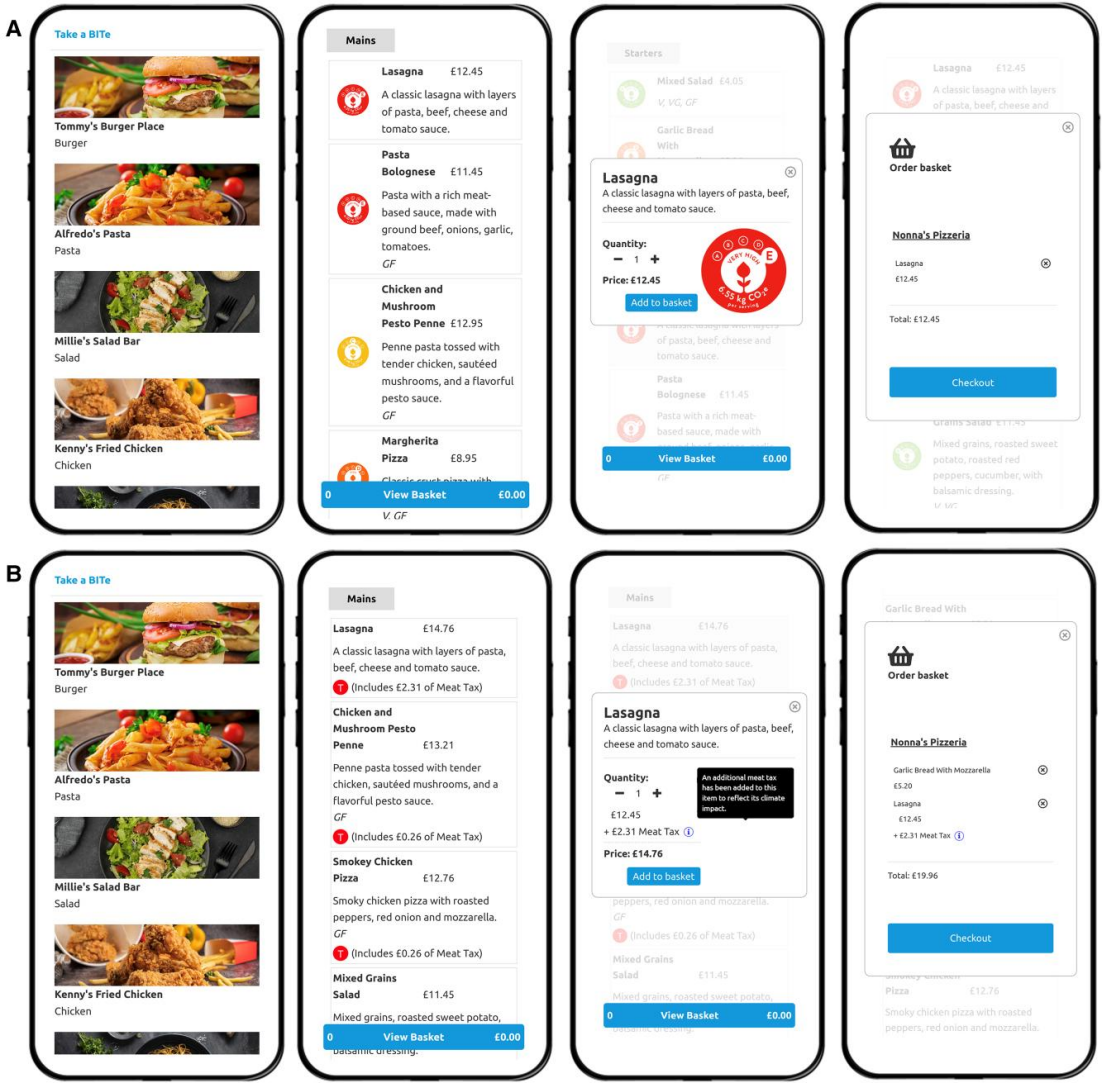
Example of the choice setting for the carbon-labeling condition (A) and meat-tax condition (B). From left to right, the participants first viewed the restaurant page, then the menu page for a chosen restaurant, followed by the selection pop-up for a chosen meal, and finally the order basket pop-up to complete their order. Participants in the repositioning and control conditions saw the same pages without carbon footprint or tax information, and both restaurant and menu pages were presented in order of increasing climate impact (repositioning condition) or randomly (control condition). See Fig. S3 for a visual representation.

Figure 1 presents an example of the food-delivery app and choice setting faced by participants (using mobile devices) for the carbon-labeling condition (A) and meat-tax condition (B). From left to right, participants initially made a choice from a range of nine restaurants, leading to the opening of the respective restaurant's menu page. Once a meal was chosen from the menu, a selection pop-up for that specific choice would appear, relaying important information from the menu (i.e. labels or added tax amounts). Then participants had the option to finalize their order using the order basket pop-up. In the behavioral (choice-architecture) condition, the menu items remained unaltered, with no labels or tax information displayed. Instead, only the order of presentation was changed (screenshots of the mobile and web versions can be found in Figs. S1–S3).

In the control condition, no label or tax information was displayed, and the restaurants and menu items were presented in random order. A detailed description of the experiment and all interventions is provided in the Supplementary material, Appendix Section 2.

Before placing an order on the simulated delivery app, participants first completed a preintervention survey that collected detailed information on food-consumption habits and preferences, experience with delivery platforms, climate and consumption attitudes, political identity, and participants’ knowledge (“literacy”) of the carbon and health impacts of food. Participants then read a short introductory text with information on the platform and food-choice task. To avoid overordering, participants were asked to order at least one main and at most one additional item within a budget of £20 for themselves for dinner. To incentivize accurate and honest behavior, participants were informed that there was a 1 in 30 chance of them actually receiving the meal chosen on the platform, with the remainder of the budget being paid out via bank transfer. After placing their order, participants completed a short postintervention survey that measured self-reported satisfaction with their food choice, factors influencing their decision, and support for a range of policies that could be implemented on online-delivery platforms (see Supplementary material, Appendix Section 4 for a detailed description of all pre and postintervention survey measures).

Study participants were recruited via Predictiv, an online experiments platform developed by the Behavioral Insights Team (see Supplementary material, Appendix Section 2.2). Only individuals who actively used delivery apps and lived in urban areas were eligible to participate. The final sample (*N* = 4,008) was largely representative of the UK internet-using urban population. Summary statistics for key socio-demographic variables are presented in Table S1. Slightly over half (52%) of participants were female with an average age of 38 and an average annual income of £35,000, and 34% held a bachelor's degree or higher. Furthermore, 32% of the sample identified more with the political left, whereas 21% identified more with the political right. The remaining 47% indicated no clear preference for either left or right. A large majority of participants were omnivorous (87%) with 79% following no particular diet and 8% stating that they were flexitarian. Only 6% of participants were vegetarian, and 2% said they were vegan. The distribution of dietary preferences in our sample is thus closely aligned with that found in recent UK-wide dietary surveys (31, 32). Moreover, we find that our randomization procedure was successful in achieving balance in socio-demographic characteristics across treatment and control groups (see Table S2). Summary statistics for all other socio-demographic and attitudinal variables employed in the heterogeneity analysis are presented in Table S3.

In total, our data contain 4,008 meal purchases, with each participant making a single purchase. On average, participants ordered 1.91 items (i.e. 91% ordered two items) and they spent £13.65 on their meal purchase (see Table S4). We found that 77% of participants used a mobile device or tablet to place their order, with the remaining 23% using the desktop site. The average energy content of the chosen food items was 1,069 Kcal, and the majority of participants were satisfied with their choice. The average carbon footprint of the basket at checkout (sum of all items) was 2.45 kg CO_2_e/serving. The majority of participants (66%) chose a meat main meal, and the largest proportion of mains (33%) had a mid-range carbon impact rating (C), followed by high-carbon alternatives (D and E).

## Results

### Main treatment effects

**Fig. 2.**
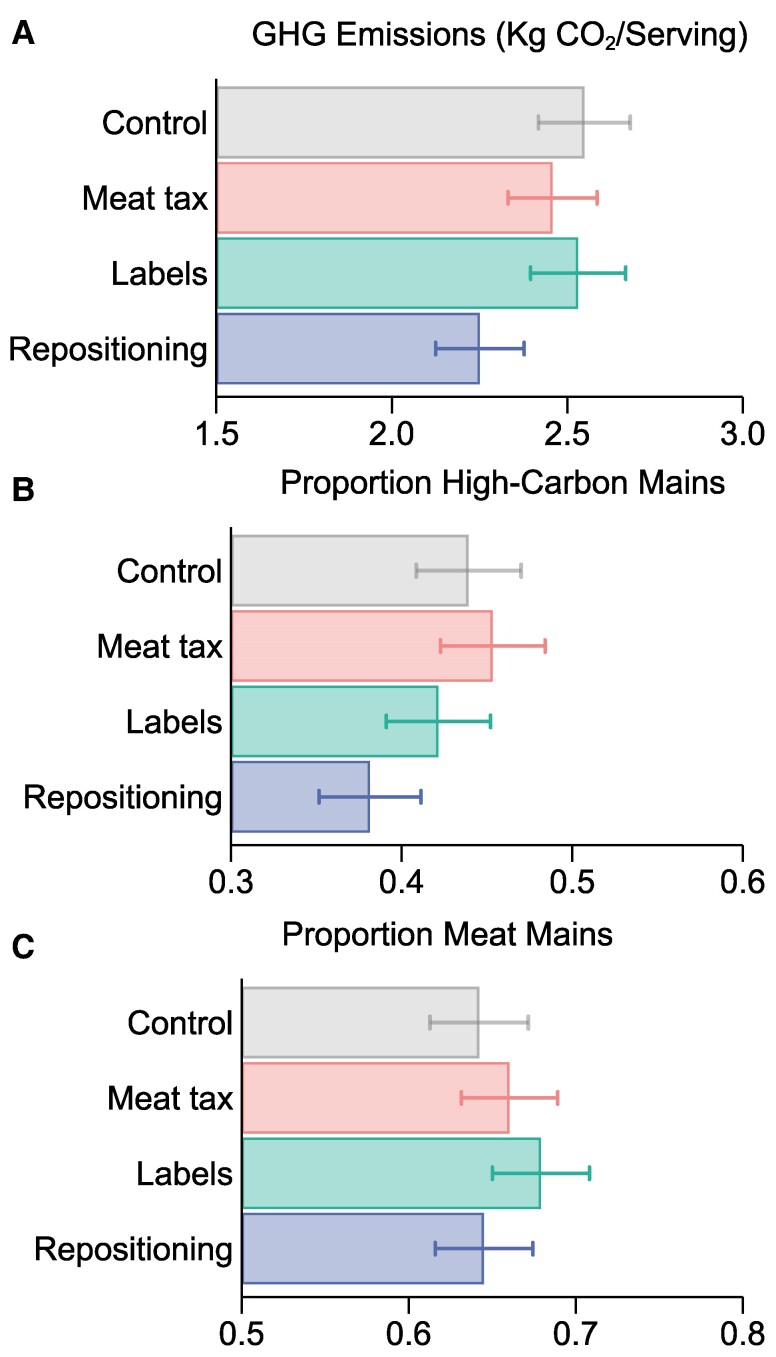
Primary outcomes. Figure displays summary statistics for sustainable food-choice outcomes in the control and three treatment conditions. Panel (A) shows the average GHG emissions (kg CO_2_e/serving) of the food basket at checkout. Panel (B) shows the proportion of high-carbon climate-impact mains chosen, and Panel (C) shows the proportion of meat mains chosen. Error bars represent 95% CIs for the unadjusted proportions. *N* = 4,008.

We first evaluate the direct impact of our three interventions on the climate impact of food choices made by all customers. Figure 2 depicts the average basket GHG emission per food purchase (A), our primary outcome of interest, as well as the proportion of high-carbon main meals (B) and meat main meals (C) purchased by treatment condition. We observe that GHG emissions were about 12% lower in the repositioning condition (2.24 kg CO_2_e/serving) compared to control (2.55 kg CO_2_e/serving), whereas emissions were only marginally lower in the meat-tax (2.47 kg CO_2_e) and carbon-labeling conditions (2.54 kg CO_2_e). We also find that participants ordered fewer high-carbon dishes in the repositioning condition (13% less relative to control) but did not significantly reduce their relative consumption of meat-based dishes. Meat dishes were chosen slightly more frequently in both the meat-tax and labeling conditions, compared to the control group. This suggests that additional interventions may be required to effectively reduce the demand for meat-based dishes in online food orders. However, despite limited impact on meat demand, the repositioning intervention did contribute to lowering the total carbon footprint significantly.

**Fig. 3.**
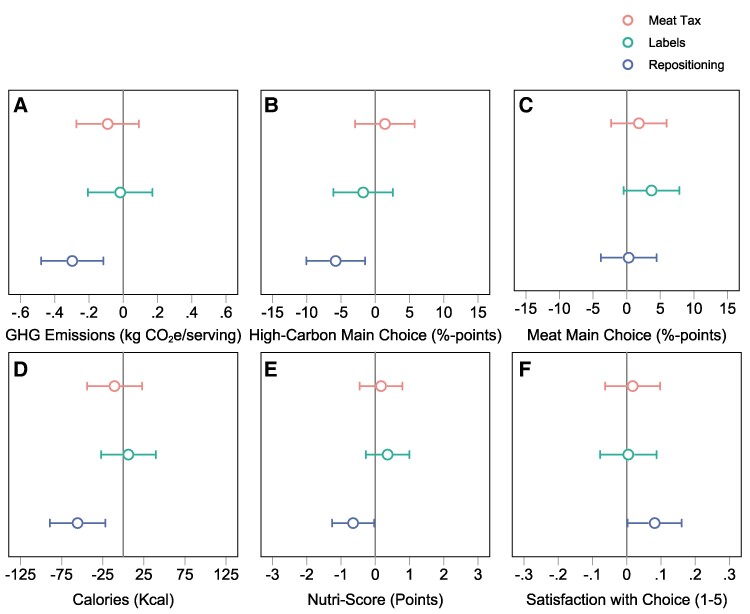
Main regression results. Figure displays the regression-estimated effects of the three treatment interventions (Meat Tax, Labels, Repositioning) on food sustainability outcomes (A–C) and health and well-being outcomes (D–F), relative to the control group. Estimates of Eq. 1 derived by OLS and LPM (see *Methods*). For ease of interpretation and visualization, point estimates provided in tile (F) are estimated by OLS. We obtain similar estimates when using an ordered probit model, which is more appropriate for ordinal (Likert-scale) response data. Specifically, we find that people were more likely to say they were “very satisfied” or “extremely satisfied” and less likely to state that they were “not at all”, “a little”, or “somewhat” satisfied. Error bars represent unadjusted 95% CIs from OLS regressions with heteroscedasticity-robust standard errors. Full regression output and randomization inference *P*-values adjusted for FWER are presented in Table S5. *N* = 4,008.

Figure 3 illustrates the average treatment effects relative to the control group obtained from estimating our preregistered model specification (see Eq. 1, *Methods*), and Table S5 presents the full regression results including *P*-values adjusted for multiple hypothesis testing. Our empirical analysis confirms that menu repositioning was the only intervention that significantly reduced the carbon impact of food choices. Menu repositioning led to a statistically significant reduction of approximately 0.3 kg CO_2_e in the average greenhouse gas emissions ordered (Fig. 3, tile A) and reduced the likelihood of choosing a high-carbon main meal by about 6 percentage points (Fig. 3, tile B), relative to the control group. However, menu repositioning had no effect on the probability of selecting a meat-based main meal.

In contrast, carbon-footprint labeling of the menu and the meat-tax intervention had minimal impact on greenhouse gas emissions of food baskets, high-carbon main selections, and meat main choices compared to the control group. As shown in Fig. 3, the estimated effects of both interventions are small and not statistically significant at the 95% confidence level for all three primary outcomes (see Supplementary material, Appendix Section 3.1 for supplementary analyses of substitution patterns between meal types). Interventions such as carbon labeling and sign-posted meat taxation strongly rely on sufficient preferences for low-carbon food consumption and are likely to be more effective if people understand and support their underlying objective (33). In line with previous research (34), taste, craving, price, and quality were the most important decision factors in our sample, and only 11% of participants stated climate impact as a decision factor when choosing their meal (see Fig. S6).

### Health and well-being co-benefits

In addition to analysing climate impacts, we examine effects on the total calories (kcal) of purchased food baskets. This is motivated by the concept of “healthy planetary diets”, which stresses that diets optimized for both human health and environmental sustainability should be a priority (3, 35–37). Figure 3, tile D, illustrates the main treatment effects and shows that the repositioning intervention significantly reduced average energy consumed by 55 kcal (or 5%), relative to the control group, decreasing the average calorie count to 1,028 kcal per basket. As calories alone represent an imperfect measure of healthfulness, we additionally computed the Nutri-Score for all dishes included on the menu (see Supplementary material, Appendix Section 2.7). Figure 3, tile E, shows that the repositioning intervention decreased the average basket Nutri-Score by 0.65 points (on a points scale ranging from −15 to +40) where lower values indicate better nutritional value, thus providing evidence of significant improvements in nutrition. For robustness, we also computed the categorized Nutri-Score (A-E rating) as well as a *Health Score* based on nutrient density (micro and macro nutrients) and USDA recommendations for a healthy diet. When using the categorized Nutri-Score (A-E) and Health Score as outcomes we find similar positive effects of repositioning on the healthfulness of food choices (see Table S5, columns 6 and 7). Both findings indicate that interventions aimed at reducing food's climate impact can also have health co-benefits by decreasing calorie consumption and improving nutrition. Future research could incorporate more comprehensive diet-quality indicators and explore synergies between environmental and nutritional goals, as called for by the healthy planetary diets paradigm.

We also examine how the interventions affected consumer choice satisfaction, an important consideration when nudging choices (38–40). We measured choice satisfaction using a 5-point Likert scale directly after the orders were placed. This approach aligns with recent studies in nudge research, which have assessed choice satisfaction at the point of decision-making, reflecting the immediate impact of interventions on perceived decision quality (41, 42). Our findings indicate that repositioning resulted in the highest average rating of 3.88 (which is significantly different from control), and meanwhile no other interventions reduced consumer satisfaction (see tile F, Fig. 3, for average treatment effects). The results should alleviate concerns that efforts to reduce food's environmental footprint come at the expense of enjoyment or happiness with meal selections. Future research should aim to measure satisfaction with the meal itself (after consumption) in addition to satisfaction with choice, to provide a more comprehensive assessment of welfare implications associated with food-delivery app interventions.

To explore additional dimensions of consumer choice satisfaction, we asked participants to rate their level of satisfaction and guilt with respect to the climate and health impact of their meal (see full question wording in Supplementary material, Appendix Section 4.2). Here, we find no significant effect of any of the interventions on climate/health satisfaction or guilt, suggesting that the increase in general satisfaction observed in the repositioning condition is unrelated to climate or health motives.

### Who is most influenced by choice architecture?

**Fig. 4.**
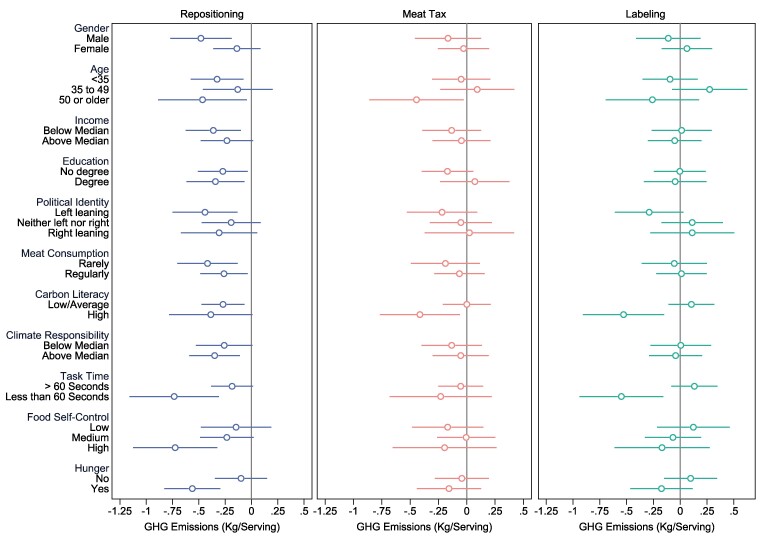
Heterogeneity analysis. Figure displays the regression-estimated effects of the three treatment interventions (Repositioning, Meat Tax, Labeling) on the average basket GHG emissions, each relative to the control condition, for specific sub-groups of the sample. Estimates of Eq. 2 obtained by OLS (see *Methods*). Error bars represent 95% CIs from OLS regressions with heteroscedasticity-robust standard errors. Full regression output presented in Table S6. *N* = 4,008.

Our results show that menu repositioning can effectively nudge more sustainable food choices. Yet individuals may vary in their responsiveness to a nudge, and a comprehensive investigation of this heterogeneity of individual characteristics can offer a more nuanced understanding of how interventions should be optimally targeted (43). We first examine whether treatment effects differ by gender, age, and socio-economic status (SES) including education and income level to identify potential equity effects for disadvantaged groups (44). To explore differences between sub-groups we estimate Eq. (2), which interacts the treatment intervention with each sub-group level (see *Methods*). Figure 4 plots the treatment effects relative to the control group for each sub-group, and Table S6 presents the full regression output including the interaction coefficient. We find that male participants were more affected by the repositioning intervention than women but observe no significant differences between age groups. Notably, we find that repositioning is effective in reducing the carbon footprint of food choices regardless of SES, with both high- and low-income/education participants responding similarly to the nudge.

Next, we explore whether repositioning has heterogeneous effects for individuals with different dietary habits, food carbon literacy, and attitudes towards individual climate action. Based on self-reported frequency of meat consumption, we identify individuals who follow a meat-heavy diet (at least three times per week) and individuals who consume meat less frequently (no more than twice a week). Moreover, we split participants into groups of low and high-carbon literacy, and self-reported personal responsibility to act on climate change. We find that all sub-groups are significantly influenced by the repositioning intervention, relative to similar individuals in the control group, and we find no significant interaction effects (see Table S6). However, as judged by the effect sizes presented in Fig. 4, people who consume less meat, have higher baseline carbon literacy, and perceive a greater responsibility to act on climate change are all more responsive to the repositioning intervention than their counterparts. This finding suggests that people with greater knowledge about the climate impact of their food consumption or who are more willing to act may be more receptive to the environmental signal sent by placing climate-friendly options first.

## Mechanisms

Finally, we look at potential (psychological) mechanisms behind menu-order effects. In particular, in online environments factors such as rushed decisions and limited attention could increase susceptibility to a nudge (24). To proxy attention of participants, we use total time spent on the food-choice task and distinguish between individuals who made their choice in less than 60 seconds (median = 41 seconds) and those who took more than a minute's time (median = 126 seconds). Furthermore, susceptibility likely depends on individual traits such as willpower and self-control, which can be framed both in terms of sustainability and health goals (45). On the one hand, consumers may hold low or high levels of *trait* self-control to resist food temptations. On the other hand, hunger or appetite may diminish executive functions, deplete cognitive resources, and ultimately diminish peoples’ momentary self-control to resist food temptations. To measure trait self-control of eating behaviors, we utilized the 5-item Self-Regulation of Eating Behaviour Questionnaire, developed and validated by Kliemann et al (46) for the UK population. Hunger was proxied based on the timing of participants’ involvement in our experiment. Specifically, we noted if it occurred around mealtime (lunch or dinner) or at other times during the day. In both cases, menu repositioning in favor of lower-carbon food may help people with lower levels of self-control to make more sustainable choices, by increasing the effort and time involved in finding unhealthier (and generally more unsustainable) options (47). We again estimate Eq. (2), which interacts the treatment indicators with each of the potential mechanism variables individually. We find evidence for all three of these potential pathways. First, we find that individuals who spent less time on the task (<60 seconds), held high levels of food self-control, or completed the survey near a mealtime (lunch or dinner) were particularly affected by the choice-architecture intervention, relative to their counterparts in the control group (see Fig. 4). We find significant interaction effects for all three sub-groups. These findings suggest that menu-repositioning nudges on online food-ordering apps may be particularly effective in reducing carbon emissions in settings where people spend little time deliberating over their choices. Contrary to our initial hypothesis, individuals with high trait self-control were more responsive possibly because the repositioned menu made it easier for them to act on their preexisting intentions without additional deliberation.

In sum, our heterogeneity analysis suggests that menu repositioning is largely effective in reducing carbon emissions from food choices, regardless of individual SES, attitudes, or intentions. We find that men were more responsive to the choice-architecture nudge and that attention, hunger, and self-control may all constitute relevant pathways through which effectiveness of this intervention can be enhanced.

### Additional heterogeneity analysis

Next, we explore whether specific sub-groups of the population are responsive to meat taxation or carbon-footprint labeling (see Fig. 4 and Table S6). Here, we find that carbon literacy—people's baseline knowledge about the carbon impact of food—appears to be an important factor in determining intervention effectiveness. For participants with higher carbon literacy and awareness, both the meat tax and labeling significantly reduced the greenhouse gas emissions of their food choices. Both interaction terms are significant at the 5 and 1% level, respectively. Interestingly, we find that for this specific sub-group of the sample, labeling leads to the largest reduction in GHG emissions relative to similar participants in the control group (−0.53 kg CO_2_e/serving), compared to meat taxation (−0.41 kg) and repositioning (−0.39 kg).

These findings suggest that even a small price change or informative label can incentivize more sustainable choices amongst a group of well-informed consumers. It is possible that the carbon label and environmental sign-posting of the meat tax acted as a form of “reminder nudge” by increasing salience of the hidden environmental costs of food consumption, thereby facilitating the consumption of more climate-friendly meals by more knowledgeable individuals. Future research could attempt to disentangle the effects of the economic incentive created by the price change induced by a meat tax and the nudge provided by the sign-posting (see e.g. (48)) for environmentally motivated fiscal interventions.

For individuals with low-carbon literacy, labels likely require more time and repetition to translate into knowledge gains, and ultimately to affect habit formation (see Supplementary material, Appendix Section 3.2 for supplementary analysis of knowledge effects of labels). In addition to carbon literacy, we find that attention appears to influence the effectiveness of labeling. Participants who spent less than 60 seconds on the task significantly reduced their GHG emissions by 0.55 kg CO_2_e/serving, relative to the control group. This effect may be due to the labels having the strongest impact initially, capturing immediate attention before other factors such as price or taste preferences begin to compete for consideration over the course of the decision-making processes. Both findings offer tentative support for the idea that labels primarily affect choices through salience rather than education (9, 49, 50), however, further research is needed to solidify these findings for food-delivery apps.

Finally, an important consideration in the evaluation of sustainable food policies is whether they may generate backlash among consumers. For instance, Ho and Page (49) find significant backlash effects to a carbon-labeling intervention among US consumers who do not believe that individuals have a moral duty to help address climate. In our sample of UK participants, we find no evidence that any of the interventions resulted in backlash effects: none of the sub-samples show a significant *increase* in GHG emissions ordered in response to the interventions. The equivalent sub-population in our dataset, with below-median perceptions of individual climate responsibility, did not significantly change their food-choice behavior in response to labeling or meat-tax interventions, though they significantly reduced emissions in the choice-architecture condition.

### Support for sustainable food policies on food-delivery platforms

Understanding public acceptance of and desire for different policies is crucial, as experimental efficacy does not always align with support among citizens and consumers (51). In our ex-post survey, we included a range of policy options that could be implemented on online food-delivery apps and asked participants to indicate their support on a scale from 1 to 5 (strongly oppose to strongly support). Fig. S5 portrays the distribution of opposition and support for all eleven policies. We find that most policies are supported by more than half of the participants. Policies that enjoy the highest levels of support are those that provide sustainability information via education campaigns or carbon labels, while repositioning options constitute the third most supported policy. More intrusive policies such as meat taxation, portion size reductions, advertising bans, and meat-reduction policies in general face the highest levels of opposition (see Fig. S5). Taken together, our findings suggest that menu repositioning is not only highly effective in reducing emissions but is also one of the most supported policies. This contrasts with meat taxation, which is neither supported nor effective in changing behavior in our experimental setting.

Next, we explore which factors influence individuals’ support for meat taxation, carbon labeling, and menu repositioning. To do so, we construct a binary variable identifying support for the respective policy by combining the response categories “support” and “strongly support”, and we use logistic regressions to model predictors of policy support. We find that exposure to the interventions did not induce support for such policies in the real world, with one notable exception: individuals randomly assigned to the labeling condition were 5%-points more likely to support carbon-footprint labeling on food-delivery platforms (significant at the 5% level), confirming recent empirical findings (52).

**Fig. 5.**
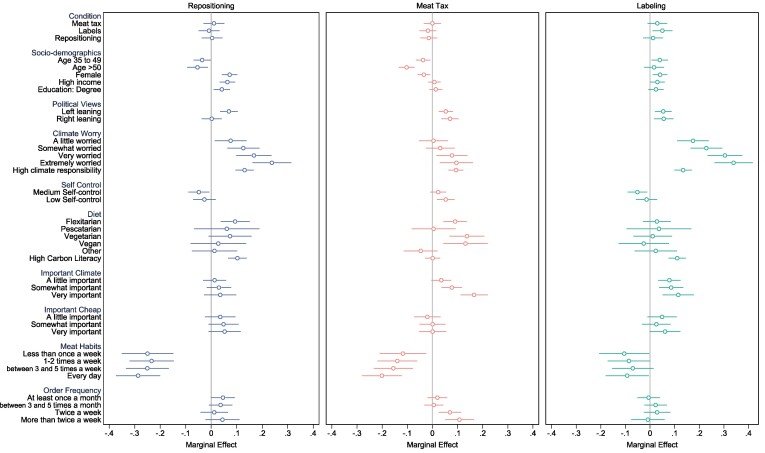
Predictors of policy support. Figure shows the predictors of policy support obtained from a logistic regression with a binary dependent variable identifying support for the respective policy (combining response categories “support” and “strongly support’). Error bars represent 95% CIs from robust standard errors. The omitted reference categories are younger than 35 (age), male and nonbinary (female), below-median income (income), no degree education (education), not at all worried (climate worry), below-median climate responsibility (climate responsibility), high self-control (self-control), none in particular (diet), not at all important that food is climate-friendly (important climate), not at all important that food is cheap (important cheap), never eat meat (meat habits), a few times a year (order frequency). Full regression estimates (marginal effects) are provided in Table S7. *N* = 4,008.

Figure 5 illustrates other predictors of policy support. A key aspect confirmed by the analysis is that individuals with meat-based diets and frequent meat eaters exhibit lower support for meat taxes, labels, and repositioning, which is expected due to their perceived self-interest. On the other hand, those who express greater concern about climate change and feel a sense of responsibility to act are more inclined to support informational interventions such as labels and repositioning in favor of climate-friendly options. Moreover, people to whom it is important that food is climate-friendly are more supportive of a meat tax and menu repositioning. Notably, we find that people with low food self-control are more supportive of meat taxation, in line with previous findings (53). However, this is not the case for labeling and repositioning. In essence, our results show that personal dietary choices and climate concerns play crucial roles in predicting support for different interventions, and in ways that align with expectations. Climate-driven consumers embrace information nudges that reinforce their environmentally friendly habits, while heavy meat eaters resist interventions that could make their preferred options more costly (including search costs).

## Discussion

The current research reveals that a simple choice-architecture intervention in an online food-delivery setting—positioning the restaurants and dishes with the smallest carbon footprint on top of the menu—can effectively nudge consumers towards more sustainable food choices, produce significant health co-benefits, and save food-related GHG emissions. The findings align with evidence from cafeteria and restaurant experiments in industrial countries (17, 18) and support the notion that plant-based defaults, with repositioning as a more subtle implementation, can reliably induce substantial changes in food choices (19, 54).

Our results reveal the relative advantage of the choice-architecture nudge over information provision (via carbon labels) and price disincentives (via meat taxation) in this specific experimental setting and are thus at odds with some field-experimental evidence (8, 10, 55). Notably, we find that labeling and meat taxation are effective only in encouraging lower-carbon food choices for people with more a priori knowledge of the climate impact of food consumption. For the average consumer, a soft order default works best.

Heterogeneity analysis suggests that menu repositioning may be particularly powerful when choices are driven by System 1 thinking (56, 57), i.e. rapid, instinctive decisions in situations where people pay little attention or give minimal deliberation to their choices (e.g. when hungry). However, with repeated exposure and more time for deliberation, individuals might adapt to repositioned platform designs by learning to scroll further down the menu to find their preferred items. To enhance long-term effectiveness, additional strategies may be needed. One promising approach involves integrating interventions that promote deliberate, System 2 thinking, such as using reflective prompts in combination with choice-architecture techniques to encourage more thoughtful and informed food choices (25). Investigating the synergies among various interventions offers another promising direction for future research (58). Future work could also explore how to better exploit digital food-choice interfaces in combination with machine learning, to learn about customers’ intentions and preferences and provide personalized and targeted interventions for healthy and more sustainable diets (59).

The findings have important implications for policy and online food retail. We show that menu repositioning offers an effective and highly accepted yet minimally intrusive strategy to support a shift towards more sustainable and healthy diets. For policymakers and decision makers on all levels (including schools and canteens), moving the more sustainable choice to the top of a menu would be a feasible, impactful, and cost-effective policy intervention. In a hypothetical scenario under which menu repositioning is implemented by the food-delivery app with the largest market share in the UK (Just Eat), with 260 million annual transactions (60), a reduction of 0.3 kg/CO_2_e on average per order would result in overall emissions savings of 78,000 metric tons per year. If estimated program costs amount to between £100,000 and £300,000 per year (considering only carbon-footprint quantification and platform developer costs), the total annual abatement cost would span from £1.28 to £3.85 per metric ton CO_2_e emissions avoided. Assuming a conservative social cost of carbon in the range of $31 to $51 (61), this would be considered highly cost-effective.

For online retail businesses and restaurants that are conscious of their brand value, our findings suggest that a simple repositioning might be seen as a positive market signal that they care about the social and environmental impact of their business and hence are contributing to solving one of the most pressing crises of today. Indeed, the food industry and retail supply chain are increasingly pressured to contribute to sustainable food systems and diets, not only for their “license to operate” but also as a show of respect for customers. At least, in our stylized setting, it does not seem to hurt financially: While we find that restaurants with the lowest-carbon menus experience financial gains under menu repositioning, we do not find a strong indication that repositioning restaurants and menus consistently harms restaurants listed further down the page (see Supplementary material, Appendix Section 3.3 for supplementary analysis of restaurant revenues). However, it is important to highlight that focusing only on the immediate financial impacts of one-time consumer purchases does not fully capture the broader economic burden for food-delivery platforms associated with menu repositioning. Implementing such a policy could potentially lead to additional economic costs for platforms, particularly those that heavily invest in optimizing item positioning algorithms to maximize profits, or rely on revenue from restaurants paying to appear at the top of the menu. A regulatory approach to implementation of online menu repositioning may thus be necessary, comparable to offline regulations, such as the UK's legislation banning High in Fat, Salt, and Sugar products from prominent retail locations to improve public health (62). While such regulations can be costly to online and offline retailers, they often yield significant long-term benefits, including enhanced sustainability and public health. Moreover, they may spur supply-side innovations, such as menu recomposition and recipe reformulation, as restaurants compete for the top spots on food-delivery apps.

Our study has several limitations that also suggest future research. First, the study does not track long-term adjustments in response to alterations of the delivery platform, as discussed above. Long-term observational studies could shed light on potential learning effects. Second, carbon labels and sign-posted meat taxation, which partially rely on information processing, might yield the desired effects only after individuals have been repeatedly exposed to them (11). Further research is necessary to comprehensively investigate dynamic effects and verify the observed null results. Third, we do not investigate the psychological mechanisms underlying menu-repositioning interventions in depth. Yet, these mechanisms are still not well understood and additional research is warranted. Fourth, our calculations of carbon saving were necessarily rough. Scaling of such an intervention would require standardized carbon-footprint calculations of all food-service providers on the platform (see Supplementary material, Appendix Section 3.4 for a detailed discussion of external validity). Fifth, the meat tax’s ineffectiveness may stem from some of our design choices, such as insufficient price increases and the £20 windfall reducing price sensitivity. Weak incentives could thus have compromised internal validity. Additionally, the tax might have acted as a moral license for more meat consumption, a hypothesis that needs to be investigated in future research. Finally, testing our hypotheses in real-world food-delivery settings, where individuals use their own money and are unaware of being observed, would enhance external validity and provide more accurate insights. Further research is needed to address these issues.

Fully realizing these limitations, our study still makes a substantial contribution to the field. It provides compelling evidence for the relative efficacy and cost-effectiveness of choice-architecture nudges on app-based food-delivery platforms to promote healthier and climate-friendlier foods without compromising customer choice satisfaction. Importantly, our heterogeneity analysis suggests that repositioning is effective regardless of SES, educational attainment, and dietary preferences, thus making it less likely to further exacerbate preexisting dietary inequalities. As in many studies on sustainable food choices, we find that women are more knowledgeable about and prone to switch towards more sustainable food choices, which calls for gender-sensitive policies and interventions (63). Taken together, our initial findings are promising and can inform decision makers on how to structure food-delivery platforms to facilitate sustainable choices. Additional strategies may be necessary to permanently steer consumer choices away from meat and animal-based options, which could further reduce the climate and environmental impacts associated with food-delivery orders.

## Methods

A detailed description of the platform design, interventions, experimental procedures, incentivization mechanism, recruitment, and data collection can be found in Section 2 (*Extended Methods*) of the Supplementary material, Appendix.

### Estimation

The primary specification used to test the three primary hypotheses is as follows:


(1) $$Y_{i}=\;\alpha +\;\beta_{1}T1_{i}+\;\beta_{2}T2_{i}+\;\beta_{3}T3_{i}+\;\gamma X_{i}+\;e_{i}$$

where $Y_{i}\;$ represents the primary outcomes of interest: $GHG_{i}$, $High_{i},$ and $Meat_{i}$. $T1_{i}$, $T2_{i}$, and $T3_{i}$ are treatment indicators equal to one if individual *i* was randomly assigned to the behavioral intervention (repositioning), information intervention (labeling), or tax intervention (meat tax) group, respectively. $X_{i}$ is a vector of socio-demographic variables for individual *i*, including age, gender, income, SES, region, and ethnicity. The model is estimated by Ordinary Least Squares (OLS), and heteroscedasticity-robust (Eicker–Huber–White) standard errors are computed.

The three preregistered primary outcomes of interest $Y_{i}$ are defined as: (i) $GHG_{i}$ stands for the GHG emission content of the food basket (sum of all items) at checkout of individual *i*; (ii) $High_{i}$ represents a binary outcome equal to one if the chosen main meal has a high carbon-footprint rating (D or E) and zero otherwise (A, B, and C); and (iii) $Meat_{i}$ represents a binary outcome equal to one if the chosen main meal is a meat dish and zero otherwise (vegan, vegetarian, or fish). The first outcome is continuous and is estimated by OLS. The latter two outcomes are binary and are estimated using linear probability models (LPM).

We address the threat of multiple hypothesis testing and the possibility of false positives by estimating randomization inference *P*-values, which were adjusted for Family-wise Error Rate (FWER) using the procedure developed by Young (64). As prespecified, we adjust for three hypothesis tests for our primary analysis (H1), which provide the main findings of this study. Moreover, we adjust for six tests for our secondary analyses (H2 and H3). FWER-adjusted *P*-values for the above tests are presented in square brackets in Table S5.

The exploratory heterogeneity analysis was conducted following Eq. (2):


(2) $$Y_{i}=\alpha +\beta_{1}T1_{i}+\beta_{2}T2_{i}+\beta_{3}T3_{i}+\gamma_{1}M1_{i}+\;\delta_{1}(M1_{i}\times T1_{i})+\delta_{2}(M1_{i}\times T2_{i})+\delta_{3}(M1_{i}\times T3_{i})+\gamma X_{i}+e_{i}$$

where M1 refers to the moderator variable of interest, which both enters as a main effect $M1_{i}$ and is interacted with the three treatment indicators ($T1_{i}$, $T2_{i}$, and $T3_{i}$). In some cases, the moderator has a third level (M2), which enters the equation in the same way as M1 but is omitted here for readability. For instance, the moderator age is a categorical variable with three levels: M0 (<35) or the omitted base category, M1 (35–49), and M2 (50 or older). We do not adjust for MHT for the exploratory analyses, as these are considered hypothesis-generating rather than confirmatory hypothesis testing.

The exploratory analysis of predictors of policy support was conducted following Eq. (3):


(3) $$P(Y_{i}=1)=\frac{1}{1+e^{-(\alpha +\beta_{1}T_{1i}+\beta_{2}T_{2i}+\beta_{3}T_{3i}+\gamma X_{i})}}$$

where $Y_{i}\;$ represents a binary outcome of policy support for meat taxation, carbon-footprint labeling, and menu repositioning (combining response categories “support” and “strongly support” from the 5-point Likert-scale response options). $T1_{i}$, $T2_{i}$, and $T3_{i}$ are treatment indicators as in Eq. (1). $X_{i}$ is a vector of socio-demographic characteristics (age, gender, income, education, political identity), climate concern (worry and responsibility to act), food self-control, food carbon literacy, frequency of food-delivery platform use, and a selection of dietary preferences (diet, meat consumption habits, importance of climate-friendly/cheap food). All models of policy support are estimated by logistic regression, and marginal effects are computed that allow estimates to be interpreted as percentage-point change in support.

## Acknowledgments

We thank Abigail Mottershaw and Bobby Stuijfzand for their support in facilitating this study and for their feedback on the design.

## Supplementary Material

pgae422_Supplementary_Data

## Data Availability

Data and code to replicate the analysis will be made publicly available via OSF repository: https://osf.io/kxh2v/.

## Preprints

This manuscript was posted on a preprint: https://ssrn.com/abstract=4818247.
