# Supplementary material for: Choice architecture promotes sustainable choices in online food-delivery apps
Source: PNAS Nexus. 2024 Sep 19;3(10):pgae422. doi: 10.1093/pnasnexus/pgae422 (PMC11450623; doi:10.1093/pnasnexus/pgae422)
Supplement: pgae422_Supplementary_Data [file pgae422_supplementary_data.pdf]

PNAS nexus

Supplementary Information for

Choice architecture promotes sustainable choices in online food-delivery apps

Paul M. Lohmann [a], Elisabeth Gsottbauer [b, c, d], James Farrington [e], Steve Human [e], Lucia A. Reisch [a]

[a] El-Erian Institute of Behavioural Economics and Policy, Judge Business School, University of Cambridge, UK; [b] Free University of Bozen-Bolzano, Competence Center of Economic, Ecological and Social Sustainability; [c] Institute of Public Finance, Department of Economics and Statistics, University of Innsbruck; [d] Grantham Research Institute for Climate Change and the Environment, The London School of Economics and Political Science, UK; [e] Behavioural Insights Team, London, United Kingdom

Paul M. Lohmann
Email: p.lohmann@jbs.cam.ac.uk

This PDF file includes:

- Figures S1 to S8
- Tables S1 to S14
- Extended Methods
- Supplementary Analyses
- Survey and Task Instructions
- SI References

Other supplementary materials for this manuscript include the following:

Replication package (data and code) available at https://osf.io/kxh2v/

1 Additional Tables & Figures

1.1 Additional figures

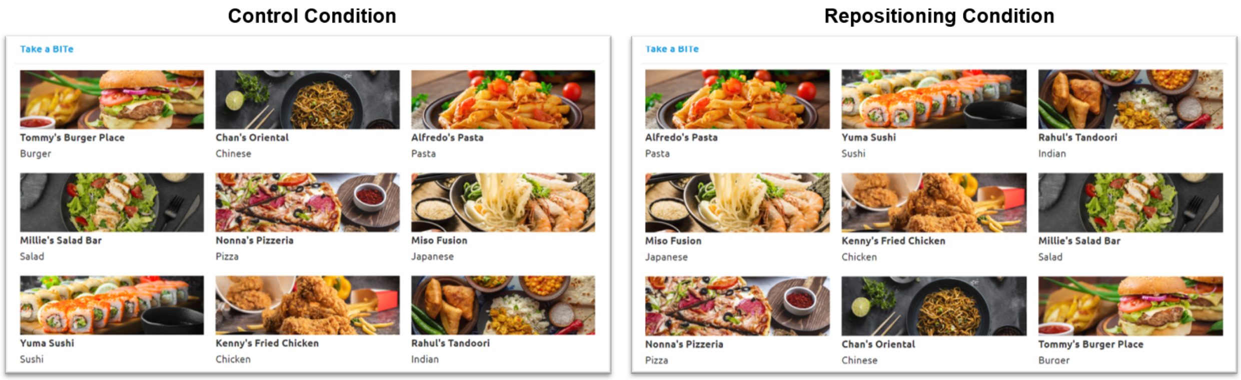

Figure S1: Web version of the restaurant page in the control condition (random order) and repositioning condition (lowest-carbon restaurants displayed first).

Control Condition **Repositioning Condition**

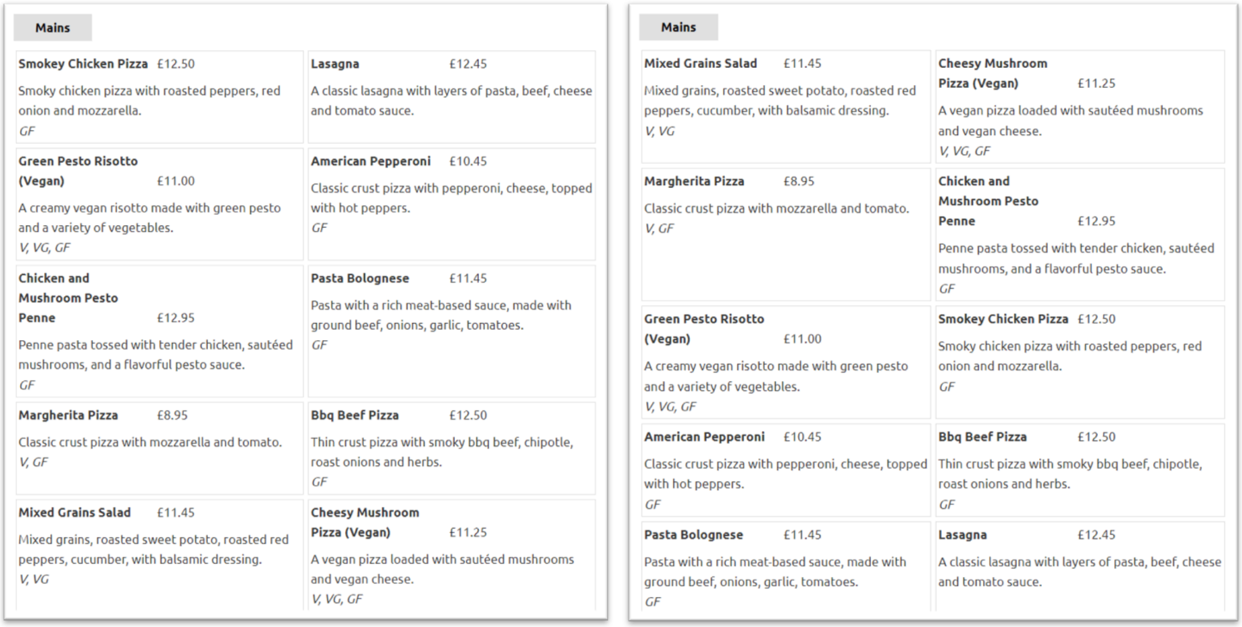

Figure S2: Web version of the menu page in the control condition (random order) and repositioning condition (lowest-carbon dishes displayed first).

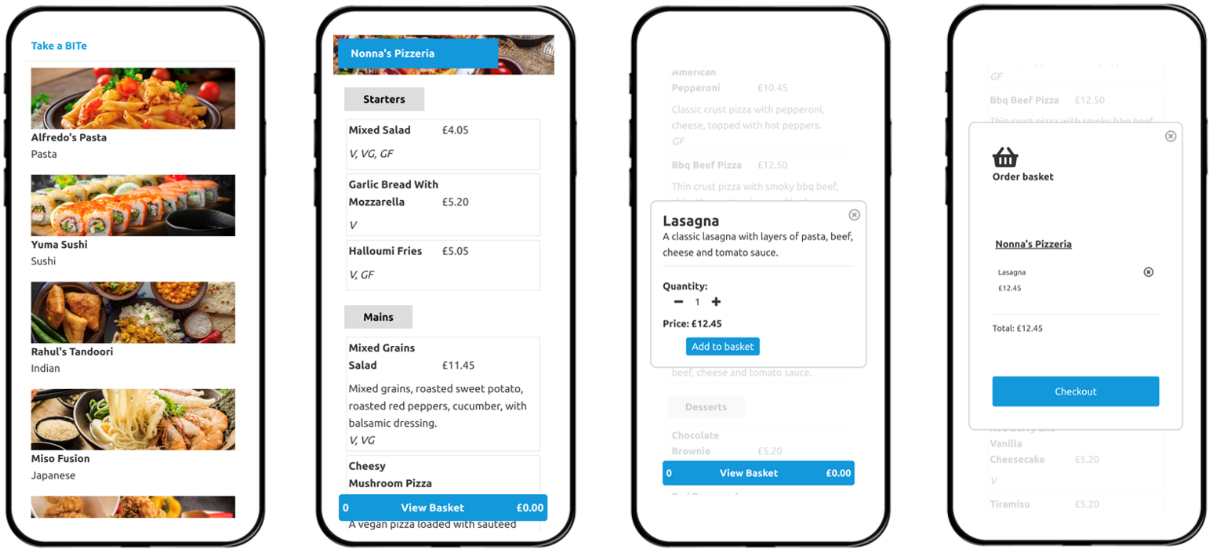

Figure S3: Mobile version of the repositioning and control condition platform layout.

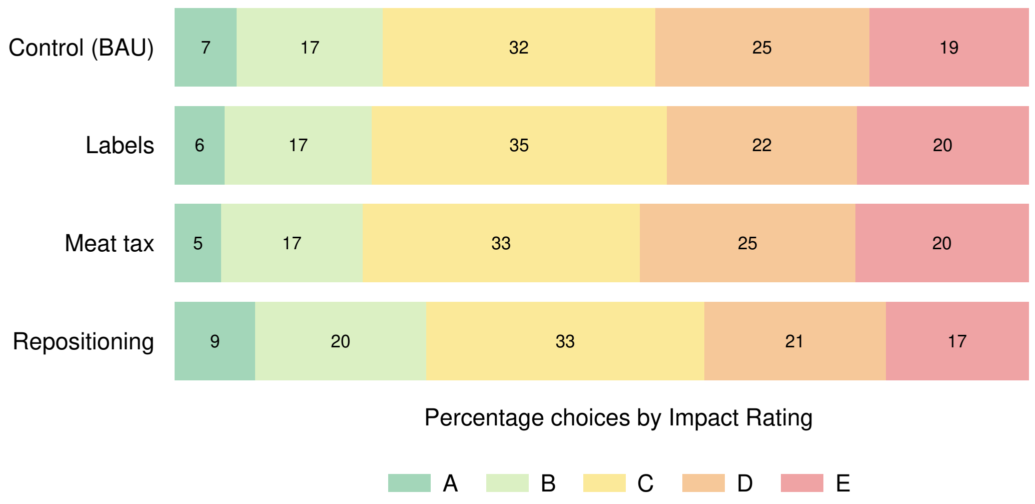

Figure S4: Percentage of mains chosen according to their climate impact score (A-E) by treatment condition.

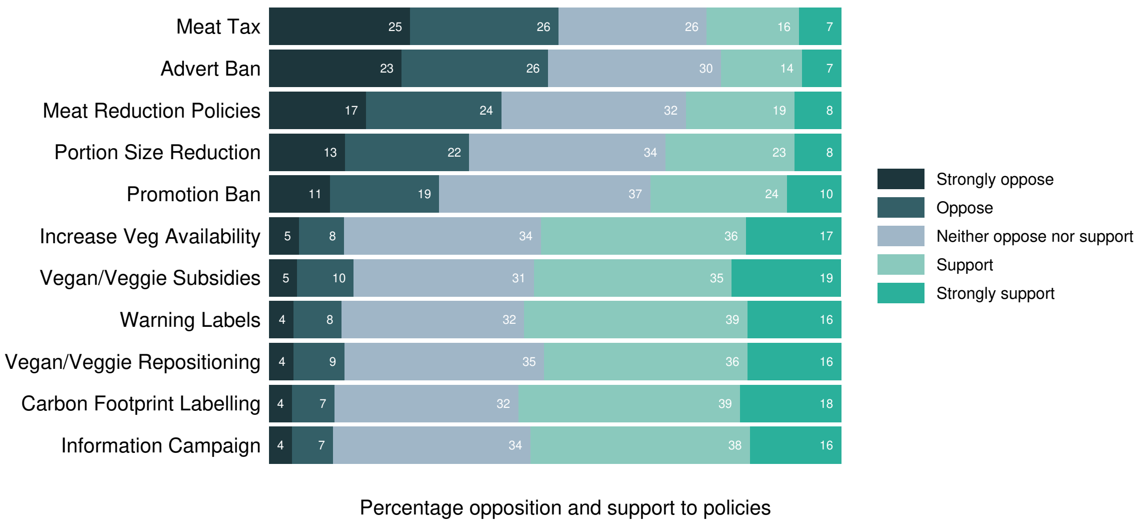

Figure S5: Support for interventions on delivery platforms. Measures on a scale from 1 to 5 from Strongly Oppose to Strongly Support.

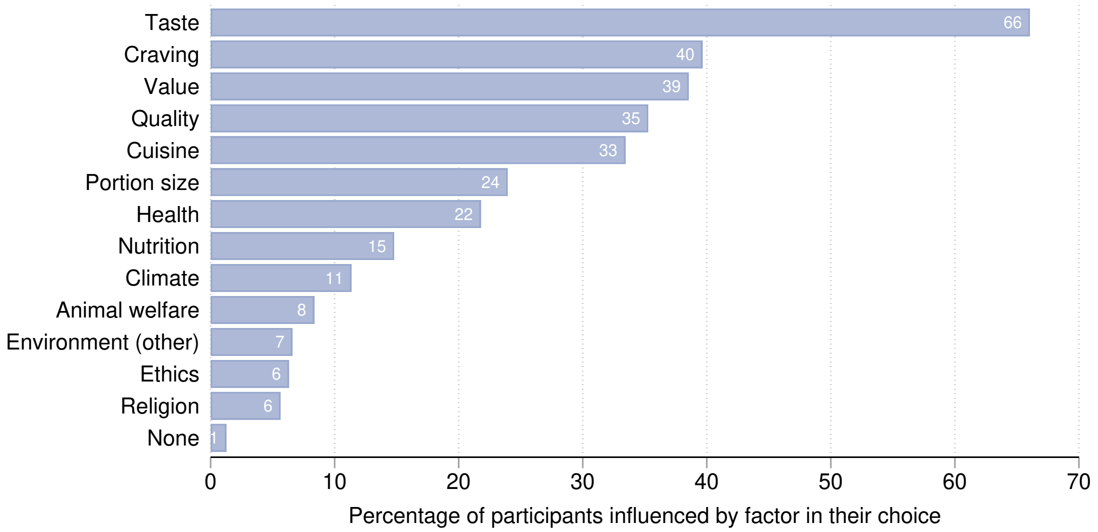

Figure S6: Decision factors.

1.2 Additional tables

Table S1: Descriptive Statistics – Full Sample

	Mean	Std. Dev.	Min	Max
Female	.52	.5	0	1
Age	37.9	13.49	18	84
Income (£ - mid-point)	35,380.46	25,465.38	2,500	100,000
Bachelor's degree or higher	.34	.47	0	1
Political identity				
Left leaning	.32	.47	0	1
Neither left nor right	.47	.5	0	1
Right leaning	.21	.41	0	1
Diet				
None in particular	.79	.41	0	1
Flexitarian	.08	.27	0	1
Pescatarian	.02	.12	0	1
Vegetarian	.06	.24	0	1
Vegan	.02	.15	0	1
Other	.03	.17	0	1

Note: $N = 4,008$.

Table S2: Descriptive statistics by group

	**Control** N=990 (24.7%)	**Meat tax** N=1,015 (25.3%)	P-value	**Labels** N=994 (24.8%)	P-value	**Repositioning** N=1,009 (25.2%)	P-value
Female	0.54 (0.50)	0.52 (0.50)	0.279	0.52 (0.50)	0.259	0.53 (0.50)	0.259
Age	38.27 (13.80)	38.10 (13.40)	0.788	37.54 (13.50)	0.233	37.68 (13.27)	0.233
Income (£)	35619.57 (25775.07)	34794.09 (25077.67)	0.467	34487.42 (24705.24)	0.318	36615.46 (26256.66)	0.318
Degree	0.35 (0.48)	0.33 (0.47)	0.212	0.33 (0.47)	0.268	0.36 (0.48)	0.268
Political identity							
Left leaning	328 (33.1%)	330 (32.5%)	0.712	319 (32.1%)	0.812	320 (31.7%)	0.812
Neither left nor right	455 (46.0%)	484 (47.7%)		471 (47.4%)		470 (46.6%)	
Right leaning	207 (20.9%)	201 (19.8%)		204 (20.5%)		219 (21.7%)	
Diet							
None in particular	765 (77.3%)	805 (79.3%)	0.681	782 (78.7%)	0.238	809 (80.2%)	0.238
Flexitarian	89 (9.0%)	79 (7.8%)		70 (7.0%)		79 (7.8%)	
Pescatarian	18 (1.8%)	19 (1.9%)		12 (1.2%)		13 (1.3%)	
Vegetarian	60 (6.1%)	57 (5.6%)		79 (7.9%)		59 (5.8%)	
Vegan	28 (2.8%)	20 (2.0%)		26 (2.6%)		22 (2.2%)	
Other	30 (3.0%)	35 (3.4%)		25 (2.5%)		27 (2.7%)	

Note: Mean (Standard deviation): P-value from pooled t-test of equality of means for the control and respective treatment group. Frequency (Percent %): P-value from Pearson Chi-squared test of equality of proportions for the control and respective treatment group.

Table S3: Summary statistics – Variables used for exploratory analysis

Variable	Mean	Std. Dev.	Min	Max
**Climate Worry**				
Not at all worried	.09	.29	0	1
A little worried	.21	.41	0	1
Somewhat worried	.3	.46	0	1
Very worried	.24	.43	0	1
Extremely worried	.15	.36	0	1
**Climate Responsibility**				
Below Median	.52	.5	0	1
Above Median	.48	.5	0	1
**Food Self-control**				
High	.17	.38	0	1
Medium	.5	.5	0	1
Low	.33	.47	0	1
**Food Carbon Literacy**				
Low/Average	.79	.41	0	1
High	.21	.41	0	1
**Important Climate**				
Not at all important	.21	.4	0	1
A little important	.32	.47	0	1
Somewhat important	.33	.47	0	1
Very important	.14	.35	0	1
**Important Cheap**				
Not at all important	.08	.27	0	1
A little important	.28	.45	0	1
Somewhat important	.4	.49	0	1
Very important	.24	.42	0	1
**Meat Consumption Habits**				
Never	.06	.24	0	1
Less than once a week	.05	.22	0	1
1-2 times a week	.18	.38	0	1
Between 3-5 times a week	.43	.49	0	1
Every day	.28	.45	0	1
**Takeaway Order Frequency**				
A few times a year	.16	.36	0	1
At least once a month	.28	.45	0	1
At least 3-4 times a month	.32	.47	0	1
Twice a week	.17	.38	0	1
More than twice a week	.08	.27	0	1
**Time Spent on Task (min)**	2.32	2.32	0.11	37.71
**Hungry**				
No	.57	.49	0	1
Yes	.43	.49	0	1

Note: Table displays the summary statistics for all additional variables not displayed in Table S1, that are used in the exploratory analysis ($N = 4,008$).

Table S4: Descriptive statistics – Food-choice task

	Mean	Std. Dev.	Min	Max
Mobile	.77	.42	0	1
Number of items	1.91	.29	1	2
Total price (£)	13.65	2.79	5.99	19.96
Total energy consumed (Kcal)	1069.24	384.95	301	1980
Satisfaction with meal choice	3.82	.91	1	5
Total basket emissions (kg co2/serving)	2.45	2.11	.11	10.22
Main meal carbon impact rating*				
A – very low carbon	.07	.25	0	1
B	.18	.38	0	1
C	.33	.47	0	1
D	.23	.42	0	1
E – very high carbon	.19	.39	0	1
Main meal type				
Meat	.66	.47	0	1

Note: $N = 4,008$. *Meals are rated based on their carbon footprint per kilogram using categories from A (Very Low) to E (Very High).

Table S5: Primary analysis

	(1) GHG	(2) High-impact Main	(3) Meat Main	(4) Calories	(5) Nutri-Score (Cont.)	(6) Nutri-Score (Cat.)	(7) Health Score	(8) Satisfaction
Meat tax	-0.091	0.014	0.018	-10.669	0.168	0.013	0.015	0.018
	(0.093)	(0.022)	(0.021)	(17.066)	(0.319)	(0.049)	(0.065)	(0.041)
	[0.354]	[0.519]	[0.363]					
Labels	-0.018	-0.018	0.037*	6.232	0.359	0.017	0.056	0.005
	(0.096)	(0.022)	(0.021)	(16.996)	(0.325)	(0.050)	(0.066)	(0.042)
	[0.846]	[0.381]	[0.075]					
Repositioning	-0.298***	-0.058***	0.003	-55.571***	-0.648**	-0.131***	0.160**	0.082**
	(0.093)	(0.022)	(0.021)	(17.225)	(0.314)	(0.048)	(0.065)	(0.040)
	[0.000]	[0.009]	[0.895]					
R^2	0.018	0.012	0.020	0.022	0.005	0.006	0.005	0.003
Observations	4,008	4,008	4,008	4,008	4,008	4,008	4,008	4,008

Note: This table presents the effect of being randomly assigned to one of three treatment groups on our primary outcomes of interest, relative to the control group. Specifically, it presents estimates of β from equation (1) estimated by OLS and LPM. In column (1), the dependent variable is total basket GHG emissions (kg CO_2e/serving). The dependent variables in columns (2) and (3) are the propensity of choosing a high-carbon or meat main dish, respectively. The remaining dependent variables are Calories (Kcal) in column (4), Nutri-Score (continuous scale from -15 to 40) in column (5), Nutri-Score (categorised scale A-E) in column (6), Health Score in column (7) and choice satisfaction in column (8). Robust standard errors in parentheses. Multiple testing adjusted P-values in square brackets based on randomisation inference (Young, 2019).

* $p < 0.1$, ** $p < 0.05$, *** $p < 0.01$.

Table S6: Heterogeneity analysis

	(1) Gender	(2) Age	(3) Income	(4) Education	(5) Politics	(6) Habits	(7) C. Lit.	(8) Clim. Resp.	(9) Task Time	(10) Self-control	(11) Hunger
Meat tax	-0.166	-0.050	-0.133	-0.170	-0.218	-0.188	0.000	-0.133	-0.054	-0.169	-0.044
	(0.149)	(0.131)	(0.132)	(0.116)	(0.159)	(0.155)	(0.108)	(0.136)	(0.101)	(0.160)	(0.122)
Labels	-0.112	-0.095	0.013	-0.004	-0.290*	-0.056	0.104	0.005	0.132	0.122	0.097
	(0.153)	(0.132)	(0.143)	(0.124)	(0.163)	(0.155)	(0.109)	(0.144)	(0.110)	(0.172)	(0.126)
Repositioning	-0.480***	-0.327**	-0.363***	-0.272**	-0.442***	-0.418***	-0.270***	-0.259*	-0.184*	-0.146	-0.099
	(0.149)	(0.128)	(0.134)	(0.121)	(0.158)	(0.146)	(0.104)	(0.137)	(0.102)	(0.171)	(0.126)
M1	-0.576***	0.076	-0.126	-0.154	-0.083	0.409***	0.155	-0.261*	0.348**	-0.170	0.163
	(0.136)	(0.147)	(0.153)	(0.136)	(0.156)	(0.139)	(0.163)	(0.134)	(0.177)	(0.151)	(0.136)
M2		0.464**			-0.180					-0.040	
		(0.186)			(0.177)					(0.199)	
M1 × Meat Tax	0.137	0.142	0.086	0.242	0.166	0.124	-0.414**	0.080	-0.177	0.163	-0.113
	(0.188)	(0.212)	(0.186)	(0.195)	(0.211)	(0.192)	(0.210)	(0.185)	(0.250)	(0.207)	(0.189)
M1 × Labelling	0.174	0.369*	-0.065	-0.044	0.401*	0.067	-0.633***	-0.048	-0.681***	-0.191	-0.272
	(0.194)	(0.222)	(0.191)	(0.193)	(0.219)	(0.196)	(0.222)	(0.191)	(0.228)	(0.219)	(0.194)
M1 × Repositioning	0.342*	0.196	0.129	-0.070	0.249	0.156	-0.116	-0.091	-0.550**	-0.088	-0.463**
	(0.187)	(0.212)	(0.185)	(0.185)	(0.213)	(0.186)	(0.228)	(0.184)	(0.239)	(0.214)	(0.186)
M2 × Meat Tax		-0.394			0.242					-0.027	
		(0.249)			(0.256)					(0.283)	
M2 × Repositioning		-0.138			0.134					-0.579**	
		(0.250)			(0.243)					(0.266)	
M2 × Labelling		-0.165			0.402					-0.292	
		(0.258)			(0.257)					(0.285)	
Female		-0.419***	-0.412***	-0.412***	-0.421***	-0.383***	-0.403***	-0.411***	-0.403***	-0.425***	-0.410***
		(0.067)	(0.067)	(0.067)	(0.068)	(0.067)	(0.067)	(0.067)	(0.068)	(0.067)	(0.067)
Age	0.010***		0.010***	0.010***	0.010***	0.011***	0.010***	0.011***	0.010***	0.011***	0.010***
	(0.002)		(0.002)	(0.002)	(0.002)	(0.002)	(0.002)	(0.002)	(0.003)	(0.002)	(0.002)
Income	-0.000	-0.000	-0.000	-0.000	-0.000	-0.000	-0.000	-0.000	-0.000	-0.000	-0.000
	(0.000)	(0.000)	(0.000)	(0.000)	(0.000)	(0.000)	(0.000)	(0.000)	(0.000)	(0.000)	(0.000)
Degree	-0.118*	-0.122*	-0.121*	0.000	-0.107	-0.117*	-0.121*	-0.100	-0.122*	-0.123*	-0.127*
	(0.070)	(0.070)	(0.070)	(.)	(0.070)	(0.070)	(0.070)	(0.070)	(0.070)	(0.070)	(0.070)
Constant	2.564***	2.742***	2.492***	2.486***	2.554***	2.137***	2.428***	2.559***	2.400***	2.535***	2.406***
	(0.152)	(0.119)	(0.143)	(0.138)	(0.165)	(0.167)	(0.134)	(0.148)	(0.139)	(0.155)	(0.138)
R^2	0.019	0.020	0.019	0.019	0.020	0.030	0.021	0.023	0.021	0.023	0.020
Observations	4,008	4,008	4,008	4,008	4,008	4,008	4,008	4,008	4,008	4,008	4,008

Note: This table presents OLS estimates of equation (2) from our heterogeneity analysis. The dependent variable is total basket GHG emissions (KG CO_2e/serving). Each column title refers to the moderator variable of interest, which is interacted with the main treatment variables. M1 refers main effect of the first level of the moderator variable. M2 refers to the second level of the moderator variable (if applicable). M0 is the omitted base category. Meat Tax, Labelling or Reordering capture the treatment effects for the omitted base catgory (e.g. in column (1) they capture the effect of the treatments for male participants). M1/M2 × Meat Tax, Labelling or Reordering, represent the interaction terms for the first/second level of the moderator with the main treatment variables. They provide the difference in the treatment effect between the omitted base category and the respective moderator level (e.g. in column (1) they capture the difference in treatment effects between male and female participants). The treatment effect of the moderator levels relative to control are not shown here, but visualised in Figure 4 in the main manuscript. Robust standard errors in parentheses.

* $p < 0.1$, ** $p < 0.05$, *** $p < 0.01$.

Table S7: Predictors of policy support

	(1) Meat Tax		(2) Carbon Labels		(3) Repositioning	
**Condition**						
Meat tax	-0.000	(0.017)	0.030	(0.020)	0.011	(0.021)
Labels	-0.018	(0.017)	0.050**	(0.020)	-0.008	(0.021)
Reordering	-0.014	(0.017)	0.012	(0.021)	0.004	(0.021)
**Socio-demographics**						
35 to 49	-0.037**	(0.014)	0.040**	(0.017)	-0.036**	(0.017)
50 or older	-0.103***	(0.016)	0.016	(0.020)	-0.054**	(0.021)
Female	-0.034***	(0.013)	0.041***	(0.015)	0.072***	(0.015)
Above Median	0.008	(0.013)	0.030**	(0.015)	0.064***	(0.015)
Degree	0.014	(0.013)	0.024	(0.016)	0.042***	(0.016)
**Political Views**						
Left leaning	0.053***	(0.014)	0.054***	(0.017)	0.069***	(0.018)
Right leaning	0.070***	(0.017)	0.056***	(0.020)	0.002	(0.020)
**Climate Worry**						
A little worried	0.004	(0.029)	0.174***	(0.033)	0.076**	(0.032)
Somewhat worried	0.031	(0.029)	0.229***	(0.033)	0.126***	(0.032)
Very worried	0.078**	(0.031)	0.304***	(0.036)	0.167***	(0.035)
Extremely worried	0.096***	(0.033)	0.339***	(0.040)	0.238***	(0.039)
Above Median	0.094***	(0.015)	0.134***	(0.018)	0.131***	(0.018)
**Self Control**						
Medium	0.023	(0.016)	-0.051**	(0.020)	-0.048**	(0.021)
Low	0.053***	(0.018)	-0.014	(0.022)	-0.027	(0.023)
**Diet**						
Flexitarian	0.090***	(0.024)	0.028	(0.029)	0.094***	(0.029)
Pescatarian	0.005	(0.044)	0.036	(0.067)	0.061	(0.065)
Vegetarian	0.138***	(0.035)	0.011	(0.040)	0.074*	(0.043)
Vegan	0.132***	(0.045)	-0.026	(0.052)	0.028	(0.055)
Other	-0.047	(0.034)	0.023	(0.044)	0.013	(0.045)
High	0.001	(0.015)	0.110***	(0.018)	0.103***	(0.019)
**Important Climate**						
A little important	0.035*	(0.020)	0.079***	(0.023)	0.013	(0.023)
Somewhat important	0.077***	(0.021)	0.086***	(0.025)	0.030	(0.024)
Very important	0.166***	(0.027)	0.115***	(0.033)	0.035	(0.032)
**Important Cheap**						
A little important	-0.020	(0.027)	0.049	(0.030)	0.035	(0.031)
Somewhat important	0.001	(0.026)	0.026	(0.030)	0.048	(0.030)
Very important	0.001	(0.027)	0.061*	(0.032)	0.053	(0.032)
**Meat Habits**						
Less than once a week	-0.118**	(0.047)	-0.105**	(0.052)	-0.250***	(0.052)
1-2 times a week	-0.140***	(0.040)	-0.086*	(0.044)	-0.234***	(0.044)
Between 3-5 times a week	-0.155***	(0.040)	-0.070	(0.043)	-0.250***	(0.043)
Every day	-0.201***	(0.040)	-0.093**	(0.045)	-0.287***	(0.044)
**Order Frequency**						
At least once a month	0.020	(0.020)	-0.006	(0.023)	0.046*	(0.024)
At least 3-4 times a month	0.006	(0.019)	0.023	(0.023)	0.037	(0.024)
Twice a week	0.070***	(0.023)	0.029	(0.027)	0.012	(0.027)
More than twice a week	0.108***	(0.029)	-0.009	(0.034)	0.044	(0.034)
Observations	4,008		4,008		4,008	

Note: This table presents the predictors of policy support obtained from a logistic regression with a binary dependent variable identifying support for the respective policy (combining response categories 'support' and 'strongly support'). Robust standard errors in parentheses. The omitted reference categories are younger than 35 (age), male and non-binary (female), below median income (income) no degree education (education), not at all worried (climate worry), below median climate responsibility (climate responsibility), high self-control (self control), none in particular (diet), not at all important that food is climate friendly (important climate), not at all important that food is cheap (important cheap), never eat meat (meat habits), a few times a year (order frequency).

* $p < 0.1$, ** $p < 0.05$, *** $p < 0.01$.

2 Extended Methods

2.1 Platform design and menu composition

We designed a delivery platform closely mirroring real-world apps like Just Eat or Deliveroo, featuring nine popular virtual restaurants including burger and chicken fast food, pizza, sandwiches and salad, sushi, pasta, Japanese, Chinese, and Indian cuisine. The cuisines represented top dining options on major UK delivery platforms. While restaurant names were generic, every restaurant's offer was modelled after a real-world counterpart, allowing us to deliver actual orders to randomly selected participants. In total, the platform offered approximately 20–25 menu items per restaurant, including main dishes, drinks, desserts, and small appetizers. Across all nine restaurants, 204 items (164 unique items) were available for order.

For this study, recipes for all menu items were developed from scratch in collaboration with Foodsteps, a company specializing in life-cycle assessment and carbon-footprint quantification. The recipes were developed to include only the most important (main) ingredients for each dish, based on meal descriptions, and thus represent an approximation of the equivalent real-world dishes. Associated carbon-footprint information and impact rating (low to high) and labels were obtained via the Foodsteps platform. Summary statistics of the average carbon footprint (kg CO2e) per serving are listed in Table S8.

Table S8: Average Carbon Footprint of Platform Menu

	Mean	Std. Dev	Min	Max	*N*
All Menu Items	1.18	1.34	0.07	9.25	164
Main Meals	1.81	1.60	0.11	9.25	78
Starters	0.81	0.78	0.07	3.61	52
Desserts	0.34	0.12	0.12	0.61	22
Drinks	0.21	0.16	0.14	0.68	12

Note: This table reports the average carbon footprint (kg CO_2e/serving) of all menu items and by menu category.

Fig. S7 displays the distribution of meals across the five impact-score categories—A (low) to E (high)—which were based on the CO2e per kg of the respective meal. Distributions of impact scores are presented for each menu category separately (mains, starters, desserts, and drinks).

Fig. S8 displays the distribution of meals across the five Nutri-Score categories—A (best) to E (worst)—which were based on nutritional values for each meal. Distributions of Nutri-Scores are presented for each menu category separately (mains, starters, desserts, and drinks).

2.2 Recruitment

Ex-ante power calculations (see pre-registration) based on data previously collected via the 'Take a BITe' platform indicated that to be able to detect a small effect size (Cohen's d = 0.15), at 80% power, with an alpha of 0.016 (Bonferroni adjusted for three hypotheses), we would require 1,000 participants per treatment arm.[1]

An online nationally representative sample of UK adults was recruited for the study through Predictiv. In total, 6,894 people started the study on Predictiv. Of these, 535 (8%) failed initial technical checks (duplicate

[1] Note that the data on which these power calculations were based differ from our final sample with respect to the final selection of restaurants and menu items included on the platform, as well as the ordering restrictions (one main + one starter/drink/dessert) imposed in the current study.

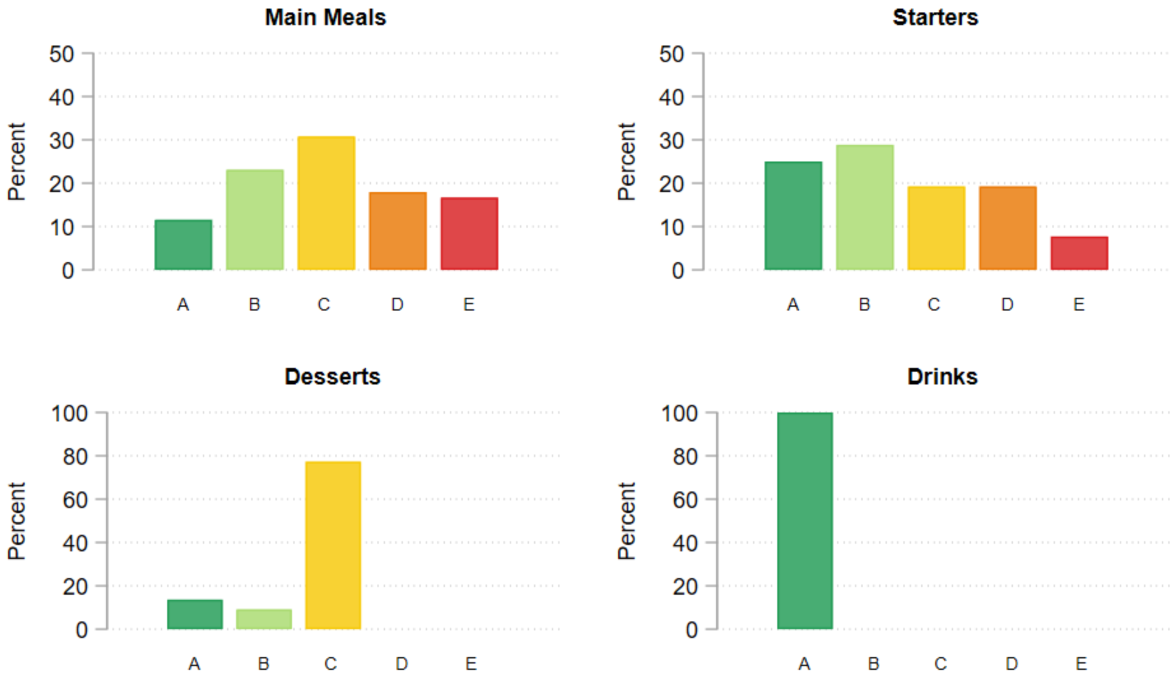

Figure S7: Climate impact score (A-E) across menu categories.

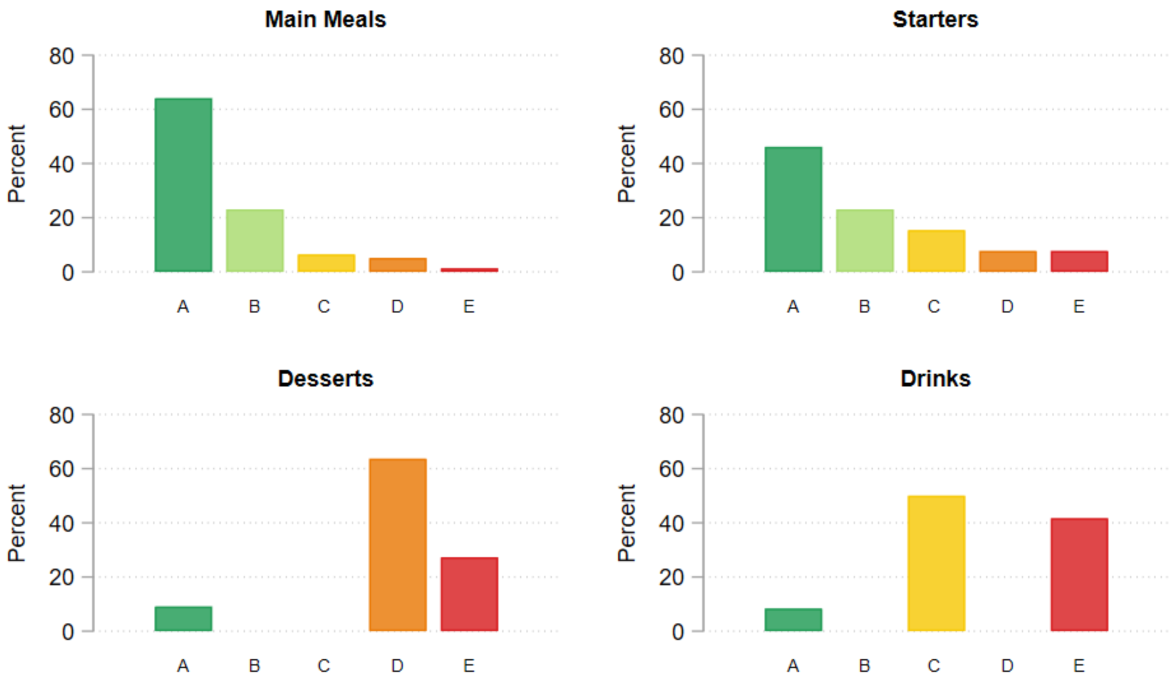

Figure S8: Nutri-Score (A-E) across menu categories.

responses, tests, or responses that had unclear/unrecognised URLs). Another 1,010 (15%) who started the study were not eligible (i.e., had never used food-delivery apps). A further 741 participants (11%) failed the attention check twice and were excluded from the study, and 312 participants (7% of those eligible and passed attention check) did not finish the study ('time-outs'). Data collection was completed after a final sample of 4,008 valid responses had been successfully recruited.

2.3 Experiment

We conducted an online randomised control trial (oRCT), which took place on the 'Take a BITe' food-delivery experimental platform. Take a BITe was developed on Predictiv by the Behavioural Insights Team (BIT).[2] The platform accurately replicated the experience of using real-life online food-delivery platforms for takeaway services, similar to popular platforms such Deliveroo, Uber Eats, and Just Eat. It featured nine restaurants that closely resemble real-world establishments and offer a diverse range of popular cuisines. Each restaurant's menu included appetizers, main dishes, desserts, and drinks (where applicable). To promote honest decision-making and discourage excessive ordering, participants were limited to choosing one main dish and one optional additional item, which could be a starter, dessert, or drink. Multiple items had to be selected from the same restaurant, and the pricing was calibrated to allow for all possible combinations to be purchased within the £20 budget at a given restaurant. We based the initial price on actual market prices as of December 2022, and prices were adjusted slightly to align with the £20 incentivization budget and to ensure a balanced selection among the restaurants. An interactive version of the platform in its standard configuration (control condition) can be previewed here.

The experiment was conducted in February 2023 with an online representative sample of 4,008 UK adults living in non-rural areas. The aim was to recruit regular users of delivery apps and ensure that the randomly selected meal choices (i.e., the experimental incentivisation) could be successfully delivered through real-world delivery platforms.

Participants had the flexibility to complete the experiment using desktop computers, tablets, or mobile devices. Prior to engaging in the food-choice task, they were asked to provide information about their dietary habits and preferences, online delivery-app use, and climate attitudes, and a self-assessment of their carbon literacy. An attention-check question was included to ensure participants' attentiveness (see SI Appendix Section 4.1). Participants who failed the attention check once were given the opportunity to adjust their response and complete the survey. Those who failed the attention check twice were not able to complete the experiment.

The subsequent food-choice task took place within an experimental environment designed to replicate online home-delivery services ('Take a BITe'). Participants were tasked with choosing a meal for dinner using a GBP 20 virtual budget, and after finishing the task, they were asked about their satisfaction with their choices, their feelings regarding their selections, factors influencing their food choice, and their recollection of any information on the webpage. To conclude, participants indicated their support for various policies and interventions related to online food-delivery platforms, such as taxes, labels, and others.

2.4 Interventions

The sample was randomly divided into four groups using a simple randomisation procedure. Approximately one-quarter of participants were assigned to the control group, which did not receive any intervention. The remaining participants were allocated to one of three treatment groups, described in detail below.

There were several elements of the platform that could be manipulated: (1) the restaurant page that showed the nine restaurants, (2) the menu page of each restaurant (visible upon selecting a restaurant), which displayed individual menu items including starters, mains, desserts, and drinks, (3) the selection pop-up that appeared upon selecting a meal, allowing users to specify the quantity of meals to be added to the basket, and (4) the checkout basket, which was visible alongside the menu page on the right of the screen (or as a

[2] https://www.bi.team/bi-ventures/predictiv/

pop-up on mobile) and which had to be reviewed before placing the order. There were four conditions: control, price intervention (meat tax), information intervention (carbon-footprint labels), and behavioural intervention (menu repositioning).

1. In the Control Condition, participants experienced the platform in its regular “business as usual” design. The restaurants and menu items were randomly ordered.

2. In the Price (Tax) Condition, the platform layout was the same as for the Control Group, with an additional tax imposed on items containing meat. The menu page of a respective item displayed a red “T” icon and the text “Includes £X.XX of meat tax.” In the selection pop-up and in the checkout basket, an extra row identified the price added by the tax as “+£X.XX meat tax.” Additional rows displayed a small information icon, providing further details about the tax’s purpose and impact (“An additional meat tax has been added to this item to reflect its climate impact”) if hovered over with the mouse or touched on a mobile device. The presentation of restaurants and menu items was randomly ordered.

   The tax was calculated for each item based on the carbon content of the meat ingredients (in kg CO2e per serving) and a carbon price of £483 per tonne of CO2e. This tax rate was chosen to achieve an average price increase of 10% across all meat dishes, equivalent to an average surcharge of 79p. The chosen tax rate aligns with research on carbon taxation in supermarkets (Panzone et al., 2018) but is below the recommendations of Funke et al. (2022), who suggest an average price increase ranging from 20% to 60% to fully account for GHG emissions and nutrient pollution related to meat production and consumption. A 10% average increase in the total price of meat dishes was deemed to reflect a more realistic scenario under which a meat tax could be introduced. The tax was ‘sign-posted’ to enhance its potential impact (Chetty et al., 2009; Gravert & Shreedhar, 2022).

3. In the Information (Labels) Condition, the platform design was identical to the Control Group, with additional information in the form of carbon-footprint labels included on the menu page of every food item and selection pop-up. The restaurants and menu items were presented in random order.

   The label design was informed by previous research (Lohmann et al., 2022; Roa-Goyes & Pickering, 2024) and executed in cooperation with our industry partner, Foodsteps. Meal items are rated based on their carbon footprint per kilogram using categories from A (very low) to E (very high), following the Global Carbon Budget for Food (2019 EAT-Lancet Commission). By choosing A-rated foods, individuals can stay within their daily carbon allowance for food.

4. In the Behavioural (Choice Architecture) Condition, no additional information was presented, but the platform design was altered to emphasize low-carbon alternatives. Both the restaurant and menu pages were re-ordered, presenting options in decreasing order of sustainability, i.e., the lowest-carbon restaurants and meal items (within each menu category) were presented first.

2.5 Incentivisation

Participants received a participation fee of £0.90 for completing the study, and they were given a virtual budget (£20) to allocate towards their online food order. It was mandatory for participants to select at least one main meal. To ensure incentive compatibility, a random incentive mechanism was employed: approximately one in thirty participants were selected at random to receive their chosen food order from the experiment, or the closest available match, after completing the study. Participants were informed about the incentivisation at the outset of the survey and directly before starting the food-choice task. Moreover, participants were informed that any remaining budget would be transferred directly to their bank account if they were chosen to receive their meal. Winners of the random incentive were subsequently contacted separately and asked to provide their address details so that the actual order could be processed and delivered on a preferred date. Alternatively, winners were given the option to donate the value of their meal to a food bank based in the UK. The research team subsequently placed the meal orders using a real-world food-delivery app. If the chosen restaurant/meal was not available in their area, a substitute meal was selected or, if no comparable substitute was available, the full value of the meal was transferred to the participant’s bank account. If there was any remaining budget from the participants’ virtual funds, it was paid out to them through an email payment service provider. Summary statistics for incentivisation implementation are shown in Table S9.

Table S9: Balance Checks

**Incentivisation Statistics**	
Winners contacted	106
No response	56
Incomplete response	5
Restaurant/Item unavailable	6
Attempted deliveries	30
Successful deliveries	27
Failed deliveries	3
Total Delivery Cost	£455.69
Total Email Payments	£420.09
Total Charity Donation	£114.92
Total Incentivisation	£990.70

Note: Incentivisation Statistics

2.6 Data and outcomes of interest

Three main outcome variables of interest were pre-registered, including (1) the sum of the carbon footprint (GHG emissions) of the meal order, (2) choices of high-carbon-impact main meals (impact score D or E), and (3) choices of meat-based main meals. Moreover, we pre-registered secondary outcome measures to explore whether the interventions influence health outcomes (i.e., calories purchased and self-reported satisfaction with the food choice), as well as support for public policies on online delivery platforms and restaurant revenue per customer. As calorie content is generally considered an incomplete measure of healthfulness, we computed the 'Nutri-Score' (Julia & Hercberg, 2017) to serve as an additional health outcome. We collected data on socio-demographic characteristics including gender, age, income, location, ethnicity, education and socio-economic status (which were merged from the Predictiv panel), political orientation, usage of food-delivery apps, climate attitudes, dietary preferences and self-control, and participants' carbon literacy in relation to food. These variables were used to conduct heterogeneity analyses. Please see Appendices 4.1 and 4.2 for a full list of survey questions. We present balance tables for key socio-demographic variables in Appendix Table S1 and summary statistics for all other socio-demographic and attitudinal variables in Appendix Table S3.

2.7 Carbon footprint and Nutri-Score calculations

Recipes were developed in collaboration with Foodsteps Ltd. We used the platform provided by Foodsteps Ltd. to calculate the carbon footprints for each meal. We used an online nutrition calculator (https://whisk.com/recipe-nutrition-calculator/) to determine macro- and micro-nutrients for each recipe. This tool was also used to calculate the Health Score. To calculate the Nutri-Score we used the official formula and code adopted from Clark et al (Clark et al., 2022). The Nutri-Score scale ranges from -15 to +40 for foods. This scale is used to assign a rating from A (best) to E (worst). The lower the Nutri-score, the higher it's nutritional value. We utilise the continuous Nutri-Score points scale in our main analysis, however, results are unchanged when using the discretized categories, See Julia and Hercberg (Julia & Hercberg, 2017) for a detailed description of the Nutri-Score, how it was derived, and how it is calculated.

2.8 Deviations from pre-analysis plan

We use a standard linear probability model (LPM) estimated by OLS to conduct confirmatory hypothesis tests of H2 and H3, instead of reporting marginal effects obtained from logistic regressions as previously pre-registered. Results are identical up to the fourth decimal place. We use the FWER multiple hypothesis testing procedure developed by Young (Young, 2019), which is more appropriate for a small number of tests,

instead of the pre-registered FDR step-down procedure (Benjamini et al., 2006).

3 Supplementary analyses

3.1 Substitution between meal types

In addition to our pre-registered outcomes of interest—choices of main meals containing meat, as detailed in the main manuscript—Table S10 presents supplementary analyses of treatment effects on vegan, vegetarian, and fish main meal choices. Estimates are obtained from equation (1) with the LPM. We find that carbon-footprint labels reduced choices of fish meals by 3.97 percentage points, relative to control. We find marginally significant positive effects (P < 0.10) of meat taxation on vegetarian main meal choices and of labelling on meat main meal choices. Similar marginal effects are observed when using a multinomial logit model to estimate substitution patterns.

Table S10: Primary Analysis Meal Type

	(1)<br>Vegan	(2)<br>Vegetarian	(3)<br>Fish	(4)<br>Meat
Meat tax	-0.0210	0.0251*	-0.0223	0.0182
	(0.0134)	(0.0141)	(0.0151)	(0.0210)
Labels	0.00438	-0.00164	-0.0397***	0.0369*
	(0.0142)	(0.0135)	(0.0147)	(0.0211)
Repositioning	0.0150	0.00551	-0.0234	0.00293
	(0.0145)	(0.0136)	(0.0152)	(0.0212)
R^2	0.009	0.007	0.010	0.020
Observations	4,008	4,008	4,008	4,008

Note: This table presents the effect of being randomly assigned to one of three treatment groups on choices of meal types, relative to the control group. Specifically, it presents estimates of β from equation (1) estimated by LPM. Depedent variables are binary indicators of vegan, vegetarian, fish, or meat main meal choices. Robust standard errors in parentheses.

3.2 Label effect on knowledge

Here, we explore whether labels were successful in increasing people's knowledge about the carbon footprint of their meal choice. During the pre-intervention survey, participants were asked to classify six different meals in terms of their carbon footprint into low, medium, or high carbon impact, which allows us to assess their level of ex ante carbon literacy. At the end of the survey, after completing their meal order, participants were asked to rate their meal choice into one of the above three categories. This variable allows us to determine whether participants correctly rated their meal, based on the actual carbon footprint and impact category of their chosen main. For the supplementary analysis presented here, we constructed a binary variable equal to one if the participant correctly rated their meal and zero otherwise. We estimate equation (1) for the full sample and sub-samples based on food carbon literacy. We find that, on average, none of the interventions significantly improved the likelihood of correctly categorising the chosen meal into low, medium, or high carbon-impact categories. However, for the segment of the sample with the lowest ex ante carbon literacy, we find that the carbon-footprint labels significantly improved correct classifications by 5.5 percentage points (or 51%), relative to the control group.

Table S11: Did Labels improve knowledge, end of survey

	(1) Full Sample	(2) Low Literacy	(3) Mid Literacy	(4) High Literacy
Meat tax	-0.018	0.008	-0.043	-0.038
	(0.015)	(0.020)	(0.028)	(0.037)
Labels	0.021	0.055**	0.000	-0.019
	(0.016)	(0.022)	(0.029)	(0.040)
Repositioning	-0.003	0.014	-0.013	-0.023
	(0.016)	(0.021)	(0.028)	(0.038)
R^2	0.004	0.008	0.004	0.003
Observations	4008	1905	1273	830

Note: This table presents the effect of being randomly assigned to one of three treatment groups on the probability of correctly categorising the carbon footprint of the chosen meal, relative to the control group. Specifically, it presents estimates of β from equation (1) estimated by LPM. Column (1) uses all observations from the full sample. Columns (2), (3) and (4) use sub-samples corresponding to low, average and high baseline carbon literacy. Robust standard errors in parentheses.

* $p < 0.1$, ** $p < 0.05$, *** $p < 0.01$.

3.3 Restaurant revenue and profit

Next, we investigate whether any of the treatment conditions led to restaurant substitution and examine the potential implications for restaurant revenue. Table S12 presents the marginal effects of the meat tax, labelling, and repositioning interventions on the choice probabilities for each of the nine restaurants available on the platform, relative to the control condition (the omitted base category). The restaurants are listed based on their average carbon footprint (shown in brackets beside each name), reflecting the order in which they were presented in the repositioning condition.

Table S12: Substitution between restaurants

	Meat Tax	Labelling	Repositioning
Pasta (0.49)	0.005	0.004	0.059***
	(0.37)	(0.33)	(4.15)
Sushi (0.74)	0.006	-0.013	0.028*
	(0.49)	(-1.04)	(2.11)
Indian (0.81)	-0.014	-0.021	-0.006
	(-0.97)	(-1.40)	(-0.36)
Japanese (0.84)	-0.013	-0.007	-0.026*
	(-1.11)	(-0.61)	(-2.38)
Fried Chicken (0.87)	-0.014	-0.003	0.007
	(-0.92)	(-0.20)	(0.46)
Salad/Sandwiches (1.12)	-0.014	-0.009	-0.020*
	(-1.46)	(-0.88)	(-2.17)
Pizza (1.18)	0.007	0.021	-0.039**
	(0.42)	(1.30)	(-2.67)
Chinese (1.22)	0.016	0.011	-0.008
	(1.06)	(0.75)	(-0.56)
Burgers (1.57)	0.022	0.016	0.005
	(1.41)	(1.05)	(0.30)

Note: Table reports marginal effect estimates of the three interventions relative to the control condition, obtained from a multinomial logit model where the dependent variable is the choice of restaurant. The restaurants are listed in order of the average carbon footprint across all items. $N = 4,008$.

* $p < 0.1$, ** $p < 0.05$, *** $p < 0.01$.

As expected, we observe that only the repositioning condition induced substitution between restaurants, and the first two restaurants that appeared at the top of the menu page (Pasta and Sushi) were more likely to be chosen. Whereas the repositioning condition decreased the choice probability of restaurants listed further down the menu (most notably Pizza), we find no effect on restaurants with the highest average carbon footprint across all meal items listed at the end of the menu (Chinese and Burgers).

An important question relates to whether substitution between restaurants resulted in different revenues and profits for the restaurants included on the platform. Table S14 displays the total quantity of meals sold and revenue earned by each restaurant under the control and treatment conditions. Although shifts between restaurants suggest that some restaurants may have benefited more than others under repositioning, substitution of sales within restaurants may limit revenue gains. Consistent with the shifts in choice probability, we observed that the two restaurants with the lowest average carbon footprints (listed first) significantly increased their revenues by £693 and £469, respectively, under repositioning compared to the control condition. We noted that both the Japanese and Pizza restaurants fared worse under repositioning (£420 and £604 loss, respectively). Nonetheless, no clear pattern emerges to support the idea that menu repositioning systematically harms restaurants listed further down the page, under the assumption that revenues accurately reflect final profits.[3]

In Appendix Table S13 we additionally explore substitution patterns within restaurants by estimating equation (1) for each restaurant individually. We find that the repositioning intervention not only induced substitution towards lower-carbon restaurants, it also reduced the average carbon footprint of ordered meals within almost all restaurants, with the exception of Japanese and Fried Chicken. This finding suggests that the combination of restaurant and menu repositioning is likely more effective than implementing either one individually, aligning with earlier research findings in the field of health and nutrition (Bianchi et al., 2023).

Table S13: Substitution within restaurants

	(1) Pasta	(2) Sushi	(3) Indian	(4) Japanese	(5) Fried Chicken	(6) Salad Sandwiches	(7) Pizza	(8) Chinese	(9) Burgers
Meat tax	-0.390**	0.036	-0.087	-0.021	0.101	-0.713*	-0.216	-0.147	-0.327
	(0.188)	(0.141)	(0.067)	(0.110)	(0.080)	(0.365)	(0.210)	(0.122)	(0.400)
Labels	-0.032	-0.313**	-0.067	0.129	0.041	-0.057	-0.215	-0.239*	-0.064
	(0.213)	(0.137)	(0.066)	(0.113)	(0.072)	(0.381)	(0.203)	(0.126)	(0.419)
Repositioning	-0.317*	-0.317**	-0.119*	0.042	0.014	-0.479	-0.357	-0.311**	-0.328
	(0.185)	(0.127)	(0.062)	(0.125)	(0.073)	(0.333)	(0.230)	(0.127)	(0.422)
R^2	0.026	0.045	0.015	0.015	0.010	0.093	0.017	0.048	0.064
Observations	417	378	502	273	606	182	547	525	578

Note: $N = 4,008$. This table presents estimates of equation (1) estimated by OLS for each restaurant sub-sample individually. The dependent variable is total basket GHG emissions (kg CO_2e/serving). Robust standard errors in parentheses.
* $p < 0.1$, ** $p < 0.05$, *** $p < 0.01$.

[3] It is important to acknowledge that profit margins may differ between menu items within the same restaurant. Here, for the purpose of simplicity, we assume that profit margins are similar for lower- and higher-carbon dishes and that restaurant profits are approximately equivalent to 6–9% of restaurant revenues.

Table S14: Sales and revenue by restaurant and condition

	Control	Meat Tax	Labelling	Repositioning
Pasta				
Total Revenue (£)	978	1090	1047	1671
Total Sales	86	93	91	147
Sushi				
Total Revenue (£)	1156	1221	963	1625
Total Sales	88	94	75	121
Indian				
Total Revenue (£)	2180	2010	1782	2092
Total Sales	135	123	113	131
Japanese				
Total Revenue (£)	1336	1116	1158	916
Total Sales	80	67	71	55
Fried Chicken				
Total Revenue (£)	1751	1762	1780	1886
Total Sales	150	143	152	161
Salad/Sandwiches				
Total Revenue (£)	596	460	490	380
Total Sales	56	43	47	36
Pizza				
Total Revenue (£)	2114	2227	2355	1510
Total Sales	138	148	159	102
Chinese				
Total Revenue (£)	1877	2250	2018	1733
Total Sales	126	145	136	118
Burgers				
Total Revenue (£)	1572	2231	1776	1602
Total Sales	131	159	150	138
Total				
Total Revenue (£)	1634	1794	1670	1649
Total Sales	990	1015	994	1009

Note: Table displays the total revenue (£) and total sales by condition for each of the nine restaurants.

3.4 External validity

To address the external validity of our findings, we adopt List's (List, 2020) SANS framework, which encompasses Selection, Attrition, Naturalness, and Scaling considerations.

First, with respect to Selection, we sampled a large representative sample of UK adults living in urban areas. The sample includes individuals who are regular users of home delivery and food-delivery platforms, which aims to ensure that our study participants closely align with the population segment that regularly utilizes these platforms. People who never used delivery platforms were not eligible to participate in the survey, which made up around 15% of all people who started the study. Moreover, recruitment was limited to urban areas, as food-delivery apps are generally not available in rural areas.

Second, while not all participants completed the study in full (due to failed technical checks, non-eligibility, failed attention checks, or time-outs), Attrition is not a concern in this study due to the one-shot between-subjects design of the experiment. Recruitment was continued until the target sample size of 4,000 participants was reached.

Third, when it comes to Naturalness, our study aimed to replicate the typical consumer experience of

interacting with an online home-delivery platform. Participants engaged in a choice task that closely resembled the process of searching for meals online, browsing through restaurant and meal options, adjusting their basket by adding or removing food items, and ultimately selecting the desired meal for purchase. Participants utilized the experimenters' funds (£20) instead of their own money, but it is worth noting that we emphasized the realistic aspect of the task by informing participants that there was a chance of actually receiving the food order they placed. Moreover, any unspent budget was transferred to the chosen participants' bank accounts, providing a real financial incentive to minimise "over-buying" on the platform. Specifically, one out of every thirty participants was randomly selected as a winner.

Fourth, with regard to Scaling, it is evident that online platforms offer a convenient and adaptable medium for implementing all the interventions (or variants of these) examined in our study. For example, such platforms could voluntarily incorporate features that allow users to sort food items based on different criteria, including sustainability and climate impact. Additionally, online platforms provide flexibility in terms of visual design and layout, making it possible to visually emphasize sustainable food options or accompany them with symbols or labels that highlight their low-carbon attributes. One limitation of implementing sustainability sorting or labelling on a webpage for food delivery is the requirement for detailed information and data on life-cycle analysis to calculate carbon footprints. However, as the demand for carbon-footprint quantification in the food industry grows, an increasing number of life-cycle assessment and footprinting services are becoming available, likely leading to reduced costs to food service providers over time.

Survey & task instructions

Pre-intervention survey

[Consumer behavior - Platform]
1. How frequently do you get an online takeaway (i.e., takeaway food order at platforms like Just Eat or Deliveroo)? [A few times a year, At least once a month, At least 3-4 times a month, Twice a week, More than twice a week]

2. Approximately how much do you spend on online takeaways each month? [£0, £10-£30, £30 - £100, £100 - £250, £250 - £500, >£500]

3. If you get an online takeaway, you usually have it... [On my own, With friends, With family, With colleagues (at work), Not sure - it varies]

[Dietary habits]
4. What diet do you follow, if any? [None in particular, Vegan, Vegetarian, Flexitarian, Pescatarian, Other [please specify].]

5. How often do you eat a sweet dessert with your main meal? [Every day, Between 3-5 times a week, 1-2 times a week, Less than once a week, Never]

6. How often do you eat food in the form of meat? (including sausage, salami, steak etc.) [Every day, Between 3-5 times a week, 1-2 times a week, Less than once a week, Never]

7. How important is it that the food you normally eat... [1: Not at all important, 2: A little important, 3: Somewhat important, 4: Very important] • is healthy? • is climate friendly? • is cheap? • is tasty?

[Self-control]
8. Please rate how the following statements apply to you from [1: Never, 2: Rarely, 3: Occasionally, 4: Frequently, 5: Always]. • I'm good at resisting tempting food. • I give up too easily on my eating intentions. • I easily get distracted from my eating intentions. • I find it hard to remember what I have eaten throughout the day. • If I am not eating in the way I intend to I make changes.

[Climate attitudes]
9. How worried are you about climate change? [1: Not at all worried, 2: A little worried, 3: Somewhat worried, 4: Very worried, 5: Extremely worried]

10. To what extent do you feel a personal responsibility to try to reduce climate change? [0: Not at all to 10: A great deal]

[Health assessment]
11. If you judge your health on a scale from 0 to 10, what would you say? [0: Very bad to 10: Excellent].

[Political ideology]
12. Some people in Britain tend to identify more with the political left, while others tend to identify more with the political right. In general, which side do you identify with more? [0: Strongly left, 2: Moderately left, 3: Slightly left, 4: Neither the left nor the right, 5: Slightly right, 6: Moderately right, 7: Strongly right].

[Carbon & health literacy]
In the following, you will see a list of 6 common restaurant meals. For each of these meals, we would like you to rate them according to how climate-friendly you think they are, as well as their calorie content. If you are unsure, give it your best guess. With climate-friendly, we are referring to the carbon footprint of a meal. Carbon footprint refers to the greenhouse gas emissions emitted during a product's life cycle including emissions from farming, packaging, processing, transport and retail. The carbon footprint of food items is calculated in line with average British consumption.

13. Considering the carbon footprint of these meals, please sort them into low, mid or high climate impact categories. [Spinach and Chickpea Curry, Spinach and Mushroom Pasta Bake, Broccoli and Stilton Quiche, Steak and Mushroom Pie, Breaded Haddock, Sweet Chilli Tofu and Vegetable Stir Fry]

With calorie content, we are referring to the calorie count of a meal. Calorie count refers to the amount of energy in food, measured in units of calories. 14. Considering the calorie content of these meals, please sort them into low, medium or high calorie categories. [Spinach and Chickpea Curry, Spinach and Mushroom Pasta Bake, Broccoli and Stilton Quiche, Steak and Mushroom Pie, Breaded Haddock, Sweet Chilli Tofu and Vegetable Stir Fry]

[Attention check]
15. People are very busy these days and some do not properly read survey questions. To show that you've read this much, answer both "Extremely interested" and "Very interested."

3.5 Post-intervention survey

[Choice factors]
1. How satisfied are you with your meal choice? ... [1: Not at all satisfied, 2: A little satisfied, 3: Somewhat satisfied, 4: Very satisfied, 5: Extremely satisfied]

2. How satisfied are you with your meal choice based on... [1: Not at all satisfied to 5: Extremely satisfied] • its climate impact? • its health (particularly caloric) impact?

3. How would you rate your level of guilt or concern about... [1: Not at all guilty/concerned, to 5: Extremely guilty/concerned] • the climate impact of your meal choice? • the caloric value and health impact of your meal choice?

4. Which factors most influenced your food choice? Select all that apply [Taste, Quality, Craving, Cuisine, Price (value for money), Ethics, Climate impact, Animal welfare concerns, Other environmental concerns (water pollution, air pollution, land use change, biodiversity loss), Portion size, Nutritional content, Health, Cultural/religious reasons, None of the above, Other]

[Policy support]

5. What do you think about the policies below for online takeaway platforms? [1: Strongly oppose, 2: Oppose, 3: Neither oppose nor support, 4: Support, 5: Strongly support]

• A tax on meat and dairy products. • Reduction in the price of meat-alternatives (vegan and vegetarian meals) via discounts and subsidies. • Labels indicating the carbon footprint of all food/meals. • Sustainable warning labels on high emission food/meals. • Ban on meat advertising. • Restriction of promotions of food/meals high in carbon emissions (e.g., meat dishes). • Information campaign on the importance of sustainable eating. • Reduction of default portion size of meat products/meals. • Making vegetarian options more visible through positioning and changing the menu order. • Decrease availability by reducing the number of available meat options. • Increase availability by increasing the number of available meat-free options.

6. How much are you willing to limit the consumption of meat and dairy products you eat? [Not at all, A little, A moderate amount, A lot, A great deal]

7. Do you recall seeing any of the following information on the menu of the restaurant you ordered from? [Yes, I recall seeing this; No, I do not recall seeing this] • Carbon footprint of the items • Calorie information for each item • Meat tax for meat items • VAT tax costs

8. How would you rate the total calorie content of the food you ordered? [Low, Medium, High]

9. How would you rate the total carbon footprint of the food you ordered? [Low, Medium, High]

3.6 Food choice task instructions

Your task:

In this next section, we want you to imagine you are ordering dinner for yourself on an online delivery platform. You will be given a virtual budget of £20 to spend on our online food delivery platform. **It is important that you make a careful choice, as there is a chance that you will actually receive the order you place.** If you are selected as a winner, we will also pay you out the remainder of your budget via bank transfer. You can use our food delivery platform just like you would in real life: you can browse through multiple restaurants, view their menus, and add or remove foods from your basket. Once you are happy with your order you can click 'checkout' to complete the task. After you have made your purchase, we will run a random draw [1 out of 30], and you will be notified whether you are selected to receive your purchase at the end of the survey. If you are a winner, we will contact you after the study by email and ask you to provide your name and address details, so that the meal can be delivered to your home. You will be able to choose from a number of dates and times for your order to be delivered. When making your choice please remember:

• You are ordering **a meal for yourself** • Spend between **£5 and £20** • Please order **at least one 'Main'** • Please order **a maximum of two items** including your main (without duplication) • Some of you will **receive your order** and any **remaining budget via bank transfer**

References

Benjamini, Y., Krieger, A. M., & Yekutieli, D. (2006). Adaptive linear step-up procedures that control the false discovery rate. *Biometrika*, *93*(3), 491–507.

Bianchi, F., Luick, M., Bandy, L., Bone, J., Kelly, S., Farrington, J., Leung, J., Mottershow, A., Murar, F., Jebb, S. A., Harper, H., & Pechey, R. (2023). The impact of altering restaurant and menu option position on food selected from an experimental food delivery platform: a randomised controlled trial. *International Journal of Behavioral Nutrition and Physical Activity*, *20*(1), 1–10.

Chetty, R., Looney, A., & Kroft, K. (2009). Salience and taxation: Theory and evidence. *American Economic Review*, *99*(4), 1145–1177.

Clark, M., Springmann, M., Rayner, M., Scarborough, P., Hill, J., Tilman, D., Macdiarmid, J. I., Fanzo, J., Bandy, L., & Harrington, R. A. (2022). Estimating the environmental impacts of 57,000 food products. *Proceedings of the National Academy of Sciences*, *119*(33), e2120584119.

Funke, F., Mattauch, L., Bijgaart, v. d. I., Godfray, H. C. J., Hepburn, C., Klenert, D., Springmann, M., & Treich, N. (2022). Toward Optimal Meat Pricing: Is It Time to Tax Meat Consumption? *Review of Environmental Economics and Policy*, *16*(2), 219–240.

Gravert, C., & Shreedhar, G. (2022). Effective carbon taxes need green nudges. *Nature Climate Change*, 8–9.

Julia, C., & Hercberg, S. (2017). Nutri-Score : Evidence of the effectiveness of the French front-of-pack nutrition label. *64*(12), 181–187.

List, J. A. (2020). Non est Disputandum de Generalizability ? A Glimpse into The External Validity Trial. (June), 1–51.

Lohmann, P. M., Gsottbauer, E., Doherty, A., & Kontoleon, A. (2022). Do carbon footprint labels promote climatarian diets? Evidence from a large-scale field experiment. *Journal of Environmental Economics and Management*, *114*(June), 102693.

Panzone, L. A., Ulph, A., Zizzo, D. J., Hilton, D., & Clear, A. (2018). The impact of environmental recall and carbon taxation on the carbon footprint of supermarket shopping. *Journal of Environmental Economics and Management*, *109*, 102137.

Roa-Goyes, S., & Pickering, G. J. (2024). Promoting a sustainable diet through carbon labeling of food: Insights from young consumers in the americas. *Sustainable Production and Consumption*, *44*, 179–187.

Young, A. (2019). Channeling Fisher: Randomization Tests and the Statistical Insignificance of Seemingly Significant Experimental Results. *The Quarterly Journal of Economics*, *134*(2), 557–598.
